# Generating highly accurate prediction hypotheses through collaborative ensemble learning

**DOI:** 10.1038/srep44649

**Published:** 2017-03-17

**Authors:** Nino Arsov, Martin Pavlovski, Lasko Basnarkov, Ljupco Kocarev

**Affiliations:** 1 Macedonian Academy of Sciences and Arts, Research Center for Computer Science and Information Technologies, Skopje, 1000, Republic of Macedonia; 2 Ss. Cyril and Methodius University, Faculty of Computer Science and Engineering, Skopje, 1000, Republic of Macedonia; 3 University of California, San Diego, BioCircuits Institute, 9500 Gilman Dr, La Jolla, CA 92093, USA

## Abstract

Ensemble generation is a natural and convenient way of achieving better generalization performance of learning algorithms by gathering their predictive capabilities. Here, we nurture the idea of ensemble-based learning by combining bagging and boosting for the purpose of binary classification. Since the former improves stability through variance reduction, while the latter ameliorates overfitting, the outcome of a multi-model that combines both strives toward a comprehensive net-balancing of the bias-variance trade-off. To further improve this, we alter the bagged-boosting scheme by introducing collaboration between the multi-model’s constituent learners at various levels. This novel stability-guided classification scheme is delivered in two flavours: during or after the boosting process. Applied among a crowd of Gentle Boost ensembles, the ability of the two suggested algorithms to generalize is inspected by comparing them against Subbagging and Gentle Boost on various real-world datasets. In both cases, our models obtained a 40% generalization error decrease. But their true ability to capture details in data was revealed through their application for protein detection in texture analysis of gel electrophoresis images. They achieve improved performance of approximately 0.9773 AUROC when compared to the AUROC of 0.9574 obtained by an SVM based on recursive feature elimination.

Machine learning has been transforming the world by improving our understanding of artificial intelligence^1^,^2^,^3^ and by providing solutions for some outstanding problems such as multi-modal parcellation of human cerebral cortex^4^ and materials discovery^5^. A learning algorithm generalizes if, given access to some training set, it returns a hypothesis whose empirical error is close to its true error^6^. There are three main approaches to institute generalization guarantees: (1) by providing bounds of various notions of functional space capacity- most notably, using the VC-dimension^7^; (2) by establishing connections between the stability of a learning algorithm and its ability to generalize^8^,^9^,^10^, and (3) by considering the compression-scheme method^11^. Here we describe an effective way to fuse boosting and bagging ensembles in which algorithmic stability directs a novel process of collaboration between the resulting ensemble’s weak/strong components that outperforms best-case boosting/bagging for a broad range of applications and under a variety of scenarios. The algorithms were assessed on various realistic datasets, showing *improved performance* in all cases, on average of slightly below 40%, compared to the best-case boosting/bagging counterparts. Furthermore, in a medical setting for protein detection in texture analysis of gel electrophoresis images^12^, our approach exhibits *surpassing performance* of approximately 0.9773 area under the ROC curve (AUROC), compared to three machine-learning feature selection approaches: Multiple Kernel Learning, Recursive Feature Elimination with different classifiers and a Genetic Algorithm-based approach with Support Vector Machines (SVMs) as decision functions, having 0.9574 or less AUROCs. Moreover, when collaboration is effectuated with weak components, our algorithm runs up to more than *five times faster* than the underlying boosting algorithm. We anticipate our approach to be a starting point for more sophisticated models for generating *stability-guided* collaborative learning approaches, not necessarily limited to boosting.

Ensemble techniques^13^,^14^,^15^ show improved accuracy of predictive analytics and data mining applications. In a typical ensemble method, the base inducers and diversity generators are responsible for generating diverse classifiers which represent the generalized relationship between the input and the target attributes. A strong classifier can be generated in probably approximately correct sense by combining weak classifiers through a procedure called boosting^16^. Boosting was the predecessor of the AdaBoost family of algorithms - which arguably became one of the most popular machine learning algorithms in recent times^17^,^18^. Bootstrap aggregating^19^, also called bagging, is a machine learning ensemble meta-algorithm designed to enhance the stability and accuracy of machine learning algorithms by reducing variance and improving overfitting. The long list of ensemble systems includes composite classifier systems^20^, mixture of experts^21^,^22^, stacked generalization^23^, combination of multiple classifiers^24^,^25^,^26^, dynamic classifier selection^27^, classifier fusion^28^,^29^, and classifier ensembles, among many others. For recent reviews on ensemble approaches for regression and classification we refer the reader to refs 30 and 31.

The basic idea of the concept of boosting is to boost the accuracy of a weak classifying tool by combining various instances into more accurate predictions. This general concept was later adapted to the field of statistical modelling, resulting into powerful methods for developing statistical models called statistical boosting algorithms: gradient boosting^32^ and likelihood-based boosting^33^. The link between statistical modelling and the original notion of boosting as a machine learning technique was established by Friedman *et al*.^34^. Later, Bühlmann and Yu^35^ used boosting algorithms to fit generalized additive regression models^36^. Boosting algorithms can be modified such that they contain an intrinsic mechanism for variable selection and model choice (component-wise learning^35^). Recently, Mayr *et al*.^37^ provide comprehensive overviews on the evolution of boosting algorithms, as well as on extending statistical boosting.

Algorithmic stability describes how a machine learning algorithm performs to small changes in the training data. In the context of modern learning theory, the use of stability can be traced back at least to the work of Rogers and Wagner^38^. The authors noted that the sensitivity of a learning algorithm with regard to small perturbations in the data controls the variance of the leave-one-out estimate and used this observation to obtain generalization bounds (w.r.t. the leave-one-out estimate) for the *k*-nearest neighbors algorithm. Kearns and Ron^39^ showed that an algorithm operating on a hypothesis class with finite VC dimension is also stable (under a certain definition of stability). Bousquet and Elisseeff^8^ introduced uniform stability and showed that it is a sufficient condition for learnability, satisfied by popular learning algorithms such as regularized linear classifiers and regressors in Hilbert spaces (including several variants of SVM). Shalev-Shwartz *et al*.^10^ considered the General Learning Setting (introduced by Vapnik) and showed that, in this setting, there are non-trivial learning problems where uniform convergence does not hold, empirical risk minimization fails, and yet they are learnable using alternative mechanisms. They identified stability as the key necessary and sufficient condition for learnability. Recently, algorithmic stability has been also connected to differential privacy^40^, (robust and perfect) generalization^6^, adaptive data analysis^41^, adaptive learning and compression schemes^42^.

In this paper we suggest a novel method for binary classification in which collaboration between (weak or strong components of) ensembles is established. Two algorithms for collaboration are suggested for which it has been proven that they are algorithmically stable. The algorithms were tested on various datasets, showing improved performance in both reducing the error rates and reducing the computation time.

## Methods

Here we focus on the problem of binary classification, i.e., positive against negative class prediction. All notations used throughout the text are summarized in Table 1. Initially, an indexed set ***x***_1_, ***x***_2_, …, ***x***_*N*_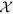 of *N* training instances and a corresponding sequence *y*_1_, *y*_2_, …, *y*_*N*_ of *N* class labels are provided. These labels are known as “binary target variables”. Assume that there is no erroneous instance labeling by any means and that each training instance has a deterministic class label in {−1, +1}. Each of the *N* training instances in 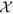 represents a *d*-dimensional real vector 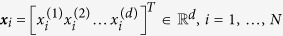, such that each vector component might have a different underlying nature and type, but can be comprehensively represented in 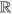. The training input to an arbitrary classification model is 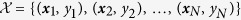, where the corresponding class label *y*_*i*_ is appended to each instance to form an input-output pair (***x***_*i*_, *y*_*i*_).

A binary classification ensemble for the data in 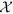 is built using *L* = *ST* weak classifiers which are grouped into *S* boosting ensembles such that each of them consists of *T* weak classifiers. Essentially, the ensemble has two levels. The first, i.e., low level consists of all weak learners, while the boosting ensembles form the second, i.e., high level. Due to this bi-level structure, an advantage is taken from both bagging and boosting throughout the model’s training^43^. The bagging technique generates multiple bootstrap samples from a single training set. The sampling variant proposed here relies on stratified sampling without replacement, resulting in multiple training subsets 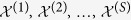, each used for learning a single boosting ensemble afterwards (details are provided in SI). Here, to illustrate our collaborative strategy we employ Gentle Boost - a variant of boosting first proposed in ref. 34 in order to make the training process terminate at a lower risk of overfitting the training data and reduce AdaBoost’s susceptibility to noise. In Gentle Boost, regression stumps are the choice for the weak learning components, introducing confidence through real-valued predictions. Let us denote each of these stumps by *f*; we add a superscript to indicate the Gentle Boost ensemble that *f* is a part of and a subscript to indicate its position (order) within that ensemble. Therefore, 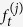 is the *t*-th regression stump in the *j*-th Gentle Boost ensemble. Given an input instance ***x***, the confidence prediction 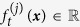 of each regression stump is a linear transformation 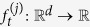, where 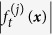 is the confidence, or strength of the prediction. The raw confidence 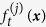 can be interpreted as a class probability using the sigmoid function *g(z*) = 1/(1 + *e*^−*z*^) (note that *g*(0) = 0.5), such that for a probability 
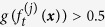
, ***x*** is labeled as positive, and otherwise, it is labeled as negative. These outputs are totaled to form the Gentle Boost ensemble’s output 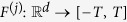, such that for any instance ***x***, each *F*^(*j*)^(***x***) can be evaluated as


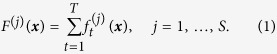


As in classical boosting, the data are weighted. We use 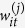 to denote the relative weight of 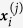 at the *t*-th iteration of boosting, with respect to the weak learner 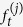. The weights are normalized such that 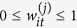 and 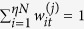 to form a probability distribution.

Finally, after all *S* Gentle Boost ensembles are trained, the totals *F*^(*j*)^(***x***) are averaged to mimic majority voting as


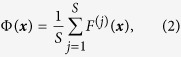


while *sign*[Φ(***x***)] is taken to be the predicted class label of ***x***. The ensemble superscript ^(*j*)^ might be left out in the following text for brevity, especially when talking about a single Gentle Boost ensemble whose position within the multi-model is unimportant. Other methods which could be incorporated in our collaborative strategy include gradient boosting and likelihood-based boosting (statistical boosting) algorithms^37^, as well as algorithms that optimize non-convex potential functions instead of the traditional exponential loss function^44^,^45^.

**Figure 1:**
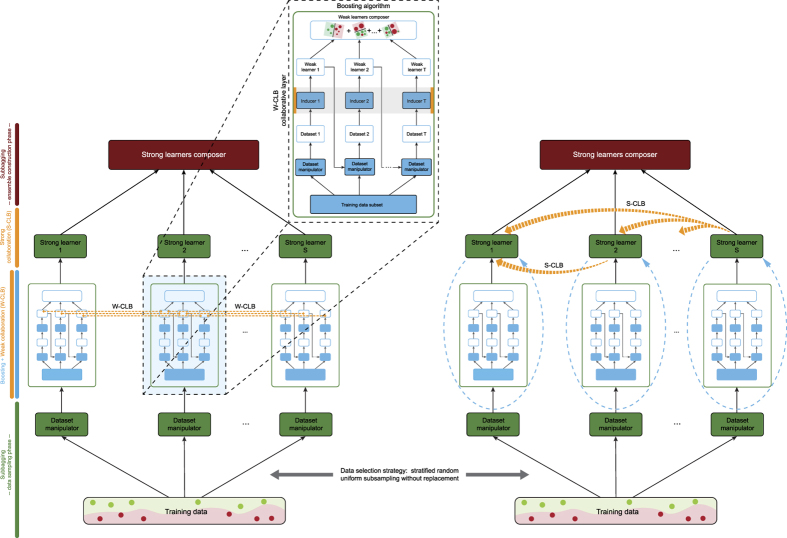
Graphical representation of the bi-level collaborative ensemble of learning machines. Being able to solve a variety of real-world problems, it automatically employs artificial collaboration (shown in orange) within two distinct levels - between the weak components of the strong machines (W-CLB, i.e., left-hand side) or between the strong machines themselves (S-CLB, i.e., right-hand side). Strong machines are constructed via the Gentle Boost algorithm, using an automatically selected subset of available domain data (shown in green and deployed by a data sampling strategy). Machine learning algorithms (specified in SI) are used by inducers to generate predictive models. W-CLB operates during boosting, while S-CLB - employed afterwards - retrains the boosting ensembles after each successful data exchange (shown by the blue dashed arrows). W-CLB and S-CLB strive to improve the constituent models’ algorithmic stability, which in turn accounts for improved performance upon integrating them into an ensemble (shown in red). The theoretical definitions of the collaborative channels (shown in grey) have been carefully designed to promote parallelization by centralizing the input data source, resulting in time-decoupled ensemble members, making them highly applicable to prodigious learning tasks.

We present two collaborative (inter-ensemble) approaches for bagging of boosting ensembles, see Fig. 1. Collaboration is carried out by exchanging instances. Both approaches aim to reduce the existing upper bounds on the overall model’s generalization error, but the key difference is the stage at which they occur. The first approach, called weak-learner collaboration (W-CLB), operates between the weak learners of different boosting ensembles; it employs collaboration during a chosen boosting round. It is a two-phase “data reorganization process”, in which the phases are interchangeably repeated at most *n*_*exc*_ times with probability *p*_*c*_ in each boosting round (see SI for description of both phases). W-CLB focuses on and penalizes correctly classified instances. More precisely, W-CLB does not perform classical margin relaxation since it replaces the instances that have been correctly predicted, with the weakest conviction. Although seemingly counterintuitive, SI makes it clear that highly confident correct predictions can be leveraged to fine-tune an optimal weak decision boundary, and most importantly, improve the overall algorithmic stability of the W-CLB approach. We provide theoretical proofs that W-CLB yields lower and potentially tighter upper bound.

The second approach, called strong-learner collaboration (S-CLB), operates only on prediction-ready ensembles, between strong boosters, after all of them have been fully trained. S-CLB is conducted through multiple consecutive iterations. At a given iteration of S-CLB, a boosting ensemble initiates a collaboration procedure, pointing to one of its predecessors within the ensemble sequence. By doing so, the initiating ensemble and the one which precedes it within the sequence form a collaboration pair that goes through three steps described in detail in SI. The procedure is repeated for all collaboration pairs of this kind. Accordingly, since the collaboration is conducted in a cumulative fashion, the multi-model’s training process consists of a number of iterations that quadratically depends on the number of boosting ensembles, but only some of them will be state-changing. An S-CLB iteration changes the model’s state only if the collaboration between a pair of ensembles at that iteration was successful. Thus, the more “jurors” comprise the “jury”, the greater the chances that some of them might collaborate. SI provides the reasons for the way S-CLB has been defined; it explains why S-CLB works and how this approach contributes to lowering and potentially tightening the model’s upper generalization error bound. Refer to SI for a fused discussion, which analyzes the procedure.

The W-CLB and S-CLB structural organizations are shown in Fig. 1(a,b), respectively. Both sub-figures recapitulate a bottom-up representation of the models’ infrastructure in terms of their building blocks along with the role of each one in the ensemble learning.

## Results and Discussion

### Theoretical results

Both the W- and S-CLB approaches employ collaboration for increasing the mean margin: boosting is a margin-maximization process that accounts for the phenomenon of generalization error reduction even after the training error reaches zero^46^. It is widely accepted that generalization performance is closely related to the increase of the margins in the training set^47^, implying lower generalization error. We use algorithmic stability to show that our collaborative approaches ensure good generalization. By extending findings in refs 48, 49, 50, 51, SI provides proofs that our collaborative strategies give lower and tighter upper bounds of the generalization error. Algorithmic stability has been utilized as reversed engineering: to guide which instances to exchange in order to improve the overall stability and reduce generalization error. The most important mathematical statements that lend the effectiveness of both collaborative strategies are presented below, while the proofs and the explanation why both strategies work are provided in the SI.

**Theorem 1.** (Generalization error upper bound of Subbagged Gentle Boost, Supplementary Theorem S6). *Assume that the loss function* 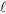 *is B-Lipschitzian, and* 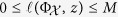
*, for all* 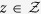
*, where* 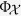 *is the outcome of a subbagging algorithm whose base machine is Gentle Boost. Next, assume that subbagging is done by sampling S sets of size p* < *N from some* 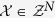 *uniformly and without replacement. Now, let the weak learning algorithm A have (pointwise) hypothesis stability β*_*w*_ *with respect to* 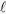 and let ε_*_ = *Weak*_*D*_(*A*)/2 > 0*. Then, for sufficiently large p, for all T, for Subbagged Gentle Boost in T rounds with probability at least* 1 − *δ over the random draw of* 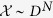,


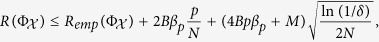


*where* 
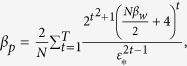



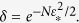


**Theorem 2.** (Classification-loss-oriented upper generalization error bound of Subbagged Gentle Boost, Supplementary Theorem S9). *Let* 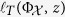 *be a T-Lipschitzian classification loss function, for all* 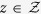
*, where* 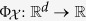 *is the outcome of a real-valued Subbagged Gentle Boost model consisted of S base Gentle Boost ensembles, while each one of them is trained using T* > 1 *weak learners. Then, for any N* ≥ 1*, and any δ* ∈ (0, 1)*, with probability at least* 1 − *δ over the random draw of a training set* 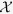,


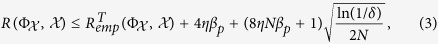


*where *β*_*p*_ is the stability of the base Gentle Boost ensemble with respect to* 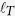, *and* 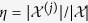.

**Theorem 3.** (W-CLB yields almost-everywhere lower empirical exponential loss of Gentle Boost, Supplementary Theorem S8). *Let t be the current round of Gentle Boost with an outcome* 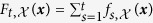 *and assume that W-CLB is injected after training* 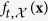
*, i.e., between rounds t and t* + 1*, yielding* 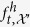 *and* 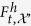
*, respectively. Then, with a high probability of ω, W-CLB yields a lower empirical Gentle Boost error* 
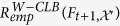
 *at round t* + 1 *with respect to the exponential loss* 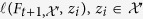
*, or*


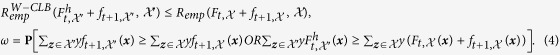


**Proposition 1.** (Supplementary Proposition S3). *Let f be the outcome of a real-valued classification algorithm, trained on a dataset* 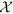 *and let* 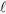 *be the exponential loss. Then for any two correctly classified training instances* 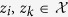, *such that* 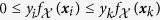,


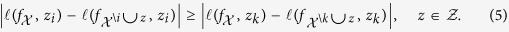


**Theorem 4.** (Monotonicity of the empirical error estimate, Supplementary Theorem S10). *Let* 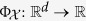 *be the outcome of a real-valued collaborative Subbagged Gentle Boost model trained on* 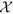
*. If S-CLB is used as a method for collaboration between its constituent Gentle Boost ensembles, then* 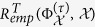
*, as a function of τ, monotonically decreases as the value of τ increments by one.*

**Proposition 2.** (Supplementary Proposition S4). *Let* 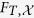 *be the outcome of a Gentle Boost algorithm trained on* 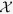 *in* *T boosting rounds that acts like a base machine of a real-valued Subbagged Gentle Boost. Then, given two positive integers* *T*′ *and* *T*′′ *such that* *T*′ ≤ *T*′′, *for any instance* 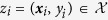 *that is correctly classified by both* 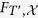 and 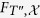,


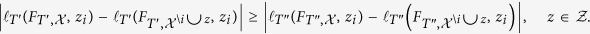


### Numerical results

We tested the algorithms on nine publicly available datasets: Australian^52^, Breast Cancer^53^, Diabetes^52^, Heart^52^, Ionosphere^52^, Liver Disorders^52^, Lung Cancer^52^, Mammographic^52^, and Vote^52^. SI provides details on the datasets, see Supplementary Table S1. The testing data was sampled uniformly and at random using 20% to 40% of the original data, depending on the amount of available data. The rest was used for training the algorithms. Of course, in a case when a test set was supplied by the source, the split procedure was omitted. The total number of weak learners varies and the results are compared for collaborative subbagged boosting using two approaches: W-CLB and S-CLB. The minimal generalization errors are summarized in Table 2. The error decreases in per cent, along with the corresponding number of prospective collaborations between the model entities that were successful are encapsulated in Tables 3 and 4, respectively. The minimal errors shown in Table 2 for Gentle Boost and Subbagging are computed through 100,000 rounds of boosting using all available training data; for Subbagging we tested all possible subset sizes, while ensemble size ranged from 2 to 1000. We report the minimal test errors.

Although easiest to predict, our algorithms decrease an already low error on Breast Cancer, thus demonstrating an ability to capture obscure patterns in data. When applied to the Subbagged Gentle Boost ensemble, both W-CLB and S-CLB account for prediction of unobserved class labels with surpassing performances. The decrease in errors for all datasets is statistically sound. In terms of computational demand, the faster W-CLB scheme in a two-level ensemble of *ST* weak learners runs up to five times faster than boosting in *T* rounds over the training set, depending on the selected parameter values, shown in Supplementary Table S2 (the influence of these parameters on the overall complexity of both approaches is illustrated in Figures S1 and S2). This number comes from the algorithmic complexity in Section S2.1. Both approaches have affirmed their robustness to variable number of ensembles *S*, thus resulting in a twisting point, that when surpassed, adding additional ensembles to the model becomes ineffective. By operating among weak learners, W-CLB introduces a negligible computational overhead. Moreover, W-CLB operates contrary to common knowledge in margin theory because it penalizes correct predictions. Common sense implies that it is best to replace the instance with the smallest negative margin (that is, the most confident wrong prediction) by an instance with a greater positive margin. In the SI we stress this phenomenon and provide several reasons to justify the actual W-CLB approach.

**Figure 2:**
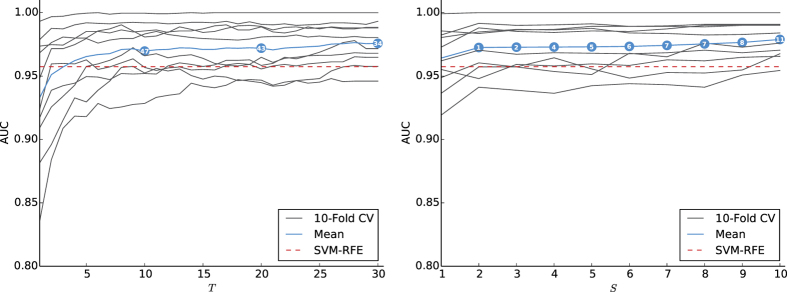
AUROC - a comparison to texture analysis in gel electrophoresis images. The line graphs show the AUROC, ranging from 0 to 1, for 10-Fold CV. The grey lines refer to the testing sets for each CV round, while the blue line is their mean. Furthermore, the red dashed line represents the highest AUROC value reported in ref. 12. The balloons on the mean curve (blue) show the number of successful collaborations throughout the model’s training in both cases, when W-CLB is used with probability *p*_*c*_ of 0.1 (left), as well as when S-CLB is the collaboration choice (right). The average AUROC over the 10 folds is 0.9758 after 30 rounds of boosting for W-CLB and 0.9789 upon adding 10 boosting ensembles for S-CLB. The best average AUROC reported in ref. 12 is 0.9574 for SVM-RFE (shown by the red dashed line), which substantiates the generalization effectiveness of our collaborative approaches.

The algorithms were also evaluated in a medical setting for protein detection in texture analysis of gel electrophoresis images^12^. Figure 2 summarizes our findings. Comparing to different machine-learning algorithms: subgroup-based Multiple Kernel Learning, Recursive Feature Elimination (RFE) with various classifiers (Naïve Bayes, SVMs, Bagged Trees, Random Forest and Linear Discriminant Analysis) and a Genetic-Algorithm-based approach with SVMs as decision functions, our algorithms show surpassing performance of approximately 0.9773 AUROC, compared to 0.9574 or less (see Fig. 2). The confidence intervals for the mean AUROC over ten folds have shown that the improvements by both W-CLB and S-CLB are statistically significant, i.e., our confidence intervals are non-overlapping compared against those reported in ref. 12. This holds true beyond 25 boosting iterations (*T* > 25) for W-CLB and beyond 6 ensembles (*S* > 6) for S-CLB. The confidence interval of the mean AUROC reported in ref. 12 is (0.9574 ± 0.0029), versus (0.9758 ± 0.0122) and (0.9789 ± 0.0102) at *T* = 30 and *S* = 10, for W-CLB and S-CLB, respectively, confirming statistically significant improvements. The methods are also robust to *T* and *S* since the confidence intervals become tighter as these parameters increase.

## Discussion

While both methods achieve similar performance, they follow a dramatically different approach. There is one critical difference that sets W-CLB apart from S-CLB. The W-CLB definition employs collaboration during boosting, while the latter requires prediction-ready boosters to conduct it. According to S-CLB, each booster is retrained after a probe exchange occurs, thus retaining margin enlargement. On the other hand, W-CLB uses a modified, further training, step in favor of time complexity. This makes W-CLB a more flexible and scalable approach in time-critical scenarios. Moreover, in SI we argue that the W-CLB collaborations can be decoupled in time, making it highly scalable. In the case of S-CLB, only two boosters constituting a collaboration pair can be decoupled in time, making it less scalable. Nevertheless, the fact that each pair of boosters is retrained right after they collaborate makes this approach a safer one when time is not critical.

## Conclusions

In statistical learning, ensemble methods have become popular as a relatively simple device to improve the predictive performance of learning algorithms. Bagging, a parallel ensemble method, is a variance reduction scheme, at least for some base procedures. On the other hand, boosting methods, which are sequential ensemble algorithms, primarily reduce the bias of the model’s base procedure. By combining bagging and boosting (parallel and sequential approaches) and by designing in-training or prediction-ready inter-boosting ensemble collaboration, we have suggested approaches which outperform subbagging and boosting in both prediction accuracy and speed. The collaborative principles presented in this manuscript were firmly supported by both the theoretical and the numerical results. Moreover, parameters of both W-CLB and S-CLB algorithms, reported in Supplementary Table S2, were easy enough to find by trial and error, barring a few cases manifesting highly imbalance class distributions. Finally, we did not apply any data preprocessing, showing the algorithms’ efficacy on and robustness to raw structured data. Since boosting has been linked to statistical estimation and additive basis expansion, which in turn, opened new perspectives of using boosting methods in many other contexts than classification (including generalized regression, density estimation, survival analysis, and multivariate analysis), we hope that this approach could also be extended to other domains in statistics. Last but not least, both approaches are seemingly equivocal in their effectiveness, regardless of the level (weak or strong) on which collaboration is leveraged. Paraphrasing Schapire and Freund^18^, who compared the general concept of boosting with “garnering wisdom from a council of fools”, we could add that “collaborative fools could generate even more wisdom”.

## Additional Information

**How to cite this article**: Arsov, N. *et al*. Generating highly accurate prediction hypotheses through collaborative ensemble learning. *Sci. Rep.* **7**, 44649; doi: 10.1038/srep44649 (2017).

**Publisher's note:** Springer Nature remains neutral with regard to jurisdictional claims in published maps and institutional affiliations.

## Supplementary Material

Supplementary Information

## Figures and Tables

**Table 1:** Notations used throughout the text (in order of appearance).

Notation	Meaning
***x***	Data instance, i.e., input
*y*	Class label, i.e., output
*z* = (***x**, y*)	Instance-label pair
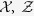	Set of instance-label pairs
*S*	Number of ensemble members
*T*	Number of boosting rounds
*f(**x***), *F(**x***), Φ(***x***)	Hypotheses, i.e., model outputs
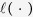	Loss function
*D*	Probability distribution
*R*(⋅)	True (generalization) error
*R*_*emp*_(⋅)	Empirical (observed) error
*β*	Algorithmic stability measure
*η*	Data subset size as a fraction
**P**[⋅]	Probability
**E**[⋅]	Expected value
*τ*	Iterator used in the collaboration context
*p*_*c*_	Collaboration probability at each boosting round
*n*_*exc*_	Number of instances to be exchanged during one collaboration

**Table 2:** Minimal generalization error rates in per cent.

Dataset	Subbagging	Gentle Boost	W-CLB	S-CLB
Australian	16.5468	12.9496	10.0719	10.7914
Breast Cancer	3.9286	3.9286	1.7857	1.7857
Diabetes	20.7792	24.0260	18.8310	18.1818
Heart	16.6667	18.5185	11.1111	11.1111
Ionosphere	8.4906	7.5472	5.6604	5.6604
Liver Disorders	20.2899	18.8406	14.4928	14.4928
Lung Cancer	2.6846	18.1208	0.6711	0.6711
Mammographic	15.0259	16.5803	14.5078	13.9896
Vote	3.4091	2.2727	1.1364	1.1364

**Table 3:** Decrease of the minimal generalization error by W-CLB and S-CLB, compared to Subbagging and Gentle Boost, respectively.

Dataset	Subbagging vs. W-CLB	Gentle Boost vs. W-CLB	Subbagging vs. S-CLB	Gentle Boost vs. S-CLB
Australian	39.13%	22.22%	34.78%	16.67%
Breast Cancer	54.55%	54.55%	54.55%	54.55%
Diabetes	9.38%	21.62%	12.50%	24.32%
Heart	33.33%	40.00%	33.33%	40.00%
Ionosphere	33.33%	25.00%	33.33%	25.00%
Liver Disorders	28.57%	23.08%	28.57%	23.08%
Lung Cancer	75.00%	96.30%	75.00%	96.30%
Mammographic	3.45%	12.50%	6.90%	15.63%
Vote	66.67%	50.00%	66.67%	50.00%

**Table 4:** Total number of successful tentative collaborations in W-CLB and S-CLB until the minimal generalization error has been reached.

Dataset	W-CLB	S-CLB
Australian	50	284
Breast Cancer	25	100
Diabetes	100	549
Heart	50	155
Ionosphere	15	107
Liver Disorders	5	390
Lung Cancer	2	149
Mammographic	120	144
Vote	83	337

The total prospective number of collaborations for W-CLB is *Tp*_*c*_*n*_*exc*_ (a successful collaboration will assure that all *S* weak learners have successfully exchanged an instance within their own training set), while this number for S-CLB is equal to *S(S* − 1)*n*_*exc*_/2.
