# Supplementary material for: Generating highly accurate prediction hypotheses through collaborative ensemble learning
Source: Sci Rep. 2017 Mar 17;7:44649. doi: 10.1038/srep44649 (PMC5356335; doi:10.1038/srep44649)
Supplement: Supplementary Information [file srep44649-s1.pdf]

Supplementary Information: Generating highly accurate prediction hypotheses through collaborative ensemble learning

Nino Arsov[1,*,+], Martin Pavlovski[1,*,+], Lasko Basnarkov[1,2], and Ljupco Kocarev[1,2,3,*]

[1]Macedonian Academy of Sciences and Arts, Research Center for Computer Science and Information Technologies, Skopje, 1000, Republic of Macedonia
[2]Ss. Cyril and Methodius University, Faculty of Computer Science and Engineering, Skopje, 1000, Republic of Macedonia
[3]University of California, San Diego, BioCircuits Institute, 9500 Gilman Dr, La Jolla, CA 92093, USA
[*]narsov@manu.edu.mk ; martin.pavlovski@cs.manu.edu.mk ; lkocarev@manu.edu.mk
[+]These authors contributed equally to this work

ABSTRACT

Ensemble generation is a natural and convenient way of achieving better generalization performance of learning algorithms by gathering their predictive capabilities. Here, we nurture the idea of ensemble-based learning by combining bagging and boosting for the purpose of binary classification. Since the former improves stability through variance reduction, while the latter ameliorates overfitting, the outcome of a multi-model that combines both strives towards a comprehensive net-balancing of the bias-variance trade-off. To further improve this, we alter the bagged-boosting scheme by introducing collaboration between the multi-models constituent learners at various levels. This novel stability-guided classification scheme is delivered in two flavours: during or after the boosting process. Applied among a crowd of Gentle Boost ensembles, the ability of the two suggested algorithms to generalize is inspected by comparing them against Subbagging and Gentle Boost on various real-world datasets. In both cases, our models obtained a 40% generalization error decrease. But their true ability to capture details in data was revealed through their application for protein detection in texture analysis of gel electrophoresis images. They achieve improved performance of approximately 0.977 AUROC when compared to the AUROC of 0.9574 obtained by an SVM based on recursive feature elimination.

Contents

S1 Supplementary Methods . . . 2
S1.1 Bagging . . . 2
S1.2 Boosting . . . 3
S1.3 Collaboration . . . 5
S1.3.1 W-CLB . . . 5
S1.3.2 S-CLB . . . 9
S1.4 Stability . . . 13
S2 Supplementary Discussion . . . 18
S2.1 Why W-CLB Works . . . 18
S2.2 Why S-CLB Works . . . 21
S3 Supplementary Data Description . . . 25
S4 Supplementary Tables . . . 26
S4.1 Parameter Value Selection . . . 26
S5 Supplementary Proofs . . . 29

S1 Supplementary Methods

S1.1 Bagging

Multiple datasets are generated by sampling independently and without replacement, at random, such that *an instance is allowed to fall into multiple subsets, but is not allowed to be duplicated (repeated) within a single subset*. Then, each resulting data subset is allotted to a boosting ensemble.

The parameters which characterize the sampling process mainly refer to the number of resources needed for its preparation, as well as their organization within the complex ensemble. In other words, these parameters conduct the preparation at first, as well as the training that follows. Each of them is listed below along with a description for its meaning and role:

- S - *Number of boosting ensembles (number of subsets)*
  Description: Denotes the number of boosting ensembles within the model, i.e., the number of generated data subsets.

- η - *Fraction of the original training set*
  Description: An approximate ratio between the cardinalities of the j-th generated subset and the original training set, where $j = 1, 2, \ldots, S$.

Note that these parameters must be set in advance. None of them is adaptive, nor one can be automatically chosen. But, although all parameters have constant values throughout the whole training, the model is not equally sensitive to all of them. For instance, the number of boosting ensembles does not play a crucial role in terms of the model's ability to generalize. On the other hand, the value of η is highly significant because it dictates the sampling process which may reflect the overall accuracy.

The sampling variant that we are proposing relies on a constraint-based independent sampling method. In fact, the main motivation for considering an independent sampling strategy is the work of Kuncheva presented on page 206[S1]. According to Kuncheva, the generation of independent samples obtains a lower generalization error compared with the case when bootstrap samples are being generated. However, in our case the original training set $\mathscr{X}$ is uniformly sampled S times without replacement, thus generating S new subsets $\mathscr{X}^{(1)}, \mathscr{X}^{(2)}, \ldots, \mathscr{X}^{(S)}$. It should be noted that instances are drawn, but not removed from the original training set while generating each subset. Additionally, every time the original training set is being sampled the following two constraints must be satisfied:

1. **Subset size constraint**
   The size of each generated subset must correspond to the predefined fraction of the original training set, denoted by η. According to this, this constraint can be represented as

   $$|\mathscr{X}^{(1)}| = |\mathscr{X}^{(2)}| = \ldots = |\mathscr{X}^{(S)}| = \eta|\mathscr{X}| = \eta N,$$

   where $\eta \in (0, 1]$. Note that when $\eta \to 1$, overlaps between some of the subsets may occur. More specifically, if $\eta = 1$, each $\mathscr{X}^{(j)}$ will represent an identical copy of the original training set. Classifiers trained on such subsets will output the same hypotheses, thus forming a totally ineffective ensemble which essentially performs identically like one of its constituent classifiers. Moreover, if instances are allowed to be duplicated within a subset, then for a large value of N the subsets $|\mathscr{X}^{(j)}|$ become bootstrap samples and the whole process refers to a bootstrap sampling process rather than to an independent one. On the contrary, when η is significantly small, some instances may not be contained in any subset, thus resulting in loss of training information. Therefore, in some cases η plays a crucial role in the process of generating subsets which contain sufficient amount of data needed for the training of each boosting ensemble.

2. **Class distribution constraint**
   According to this constraint, the class distribution of $\mathscr{X}^{(j)}$ must be preserved in accordance with the one of the original training set $\mathscr{X}$, i.e.,

   $$\frac{\sum_{i=1}^{N} \mathbb{1}_{\mathbf{x}_i \in \mathscr{X}^{(j)}} \mathbb{1}_{y_i=-1}}{\sum_{i=1}^{N} \mathbb{1}_{\mathbf{x}_i \in \mathscr{X}^{(j)}} \mathbb{1}_{y_i=1}} \approx \frac{\sum_{i=1}^{N} \mathbb{1}_{y_i=-1}}{\sum_{i=1}^{N} \mathbb{1}_{y_i=1}},$$

   where $\mathbb{1}_A$ is an indicator function which returns 0 or 1 depending on whether the event A is an impossible event or a sure one, respectively. It is implied that this must be met for all $j = 1, \ldots, S$. The need of introducing this constraint lies in the possibility of some ensembles to become biased towards instances from a certain class. By just sampling S times without replacement there is a possibility that some instances will occur in several subsets. Moreover, if the sampling is done totally random and without any restrictions, then the class distribution of some subsets might turn out to be highly homogeneous. This is usually the case when the class distribution in the original training set is imbalanced. Therefore, this constraint will not allow a subset whose class distribution is not proportional to the one in $\mathscr{X}$ to be generated. In other words, each $\mathscr{X}^{(j)}$ will represent a stratified random sample from the empirical distribution of $\mathscr{X}$.

S1.2 Boosting

Boosting is an active area of research, hence various boosting flavours have been proposed. Each of these boosting variants is implemented by a particular boosting algorithm, although boosting algorithms can be seen as specific modifications of the original AdaBoost. Some of them are specialized for binary classification, such as the binary classification variant of AdaBoost.M1, LogitBoost, GentleBoost, and RobustBoost. On the other hand, AdaBoost.M2 is intended only for multiclass classification problems. Of course, there are some algorithms eligible for performing both tasks (LPBoost, TotalBoost, RUSBoost, etc.).

Gentle Boost

This variant of boosting was first proposed in[S2] in order to reduce overfitting to the training data and susceptibility to noise. Gentle Boost's key difference from AdaBoost is the individual error function ε_t minimized by each base learner. The weighted sum of squared errors (WSSE), defined as

$$\varepsilon_t = \sum_{i=1}^{N} w_{it} \left(y_i - f_t(\boldsymbol{x}_i)\right)^2, \quad t = 1, \ldots, T, \tag{1}$$

takes all instances into account, both correctly classified and misclassified, instead of only the latter, as in AdaBoost, which previously introduced a risk of overfitting by solely focusing on the weighted, raw misclassification rate. The WSSE also implies that all predictions made by the weak learners have a certain degree of confidence since the output is not discrete, but rather real, which makes the algorithm more flexible and intuitive. Empirical evidence by Friedman has suggested that Gentle Boost, as a more conservative algorithm, has similar performance to both the Real AdaBoost and Logit Boost algorithms, and often outperforms them, especially when stability is an issue[S2].

Algorithm 1 Gentle Boost Algorithm, page 353[S2]

procedure GENTLE BOOST(input $\mathscr{X} \in \mathscr{X}^N$, class labels $\boldsymbol{y}$, number of iterations T)
  Let there be N training samples $z_1 = (\boldsymbol{x}_1, y_1), z_2 = (\boldsymbol{x}_2, y_2), \ldots, z_N = (\boldsymbol{x}_N, y_N)$.
  Start with uniform weights $w_{i,1} = 1/N$, $i = 1, \ldots, N$, and $F_{\mathscr{X}}(\boldsymbol{x}) = 0$.
  for $t = 1, 2, \ldots, T$ **do**
    Fit the regression function $f_{t,\mathscr{X}}(\boldsymbol{x})$ by weighted least-squares of each y_i to $\boldsymbol{x}_i$ with weights $w_{i,t}$.
    Update $F_{\mathscr{X}}(\boldsymbol{x}) \leftarrow F_{\mathscr{X}}(\boldsymbol{x}) + f_{t,\mathscr{X}}(\boldsymbol{x})$.
    Update the weights $w_{i,t+1} \leftarrow w_{i,t} \exp\left[-y_i f_{t,\mathscr{X}}(\boldsymbol{x}_i)\right]/Z_t$, where Z_t is a normalization constant that makes $\sum_{i=1}^{N} w_{i,t+1} = 1$.
  end for
  Output the final classifier $\operatorname{sign}\left[F_{\mathscr{X}}(\boldsymbol{x})\right] = \sum_{t=1}^{T} f_{t,\mathscr{X}}(\boldsymbol{x})$[†]
end procedure

[†] We slightly changed the definition of the voting output in order to use confidence-based predictions on all levels of the complex ensemble.

Weak Learning

A binary classifier is represented by a map $f : \mathbb{R}^d \to \{-1, 1\}$ which maps an input instance to a class label. The Vapnik-Chervonenkis statistical learning theory suggests that an effective classifier meets three conditions: (a) The classifier is trained on an adequate and sufficient amount of data; (b) The classifier demonstrates a low misclassification rate on the training data; and (c) The classifier is simple. The sampling method described in Section S1.1 satisfies only condition (a). Conditions (b) and (c) are met by the *hypothesis of weak learning - a classification model is a weak learner if it demonstrates a misclassification rate lower than* $1/2$ *and predicts the class labels more accurately than random guessing*, or formally:

Supplementary Definition S1 (Weak learner)**.** *Assume that a classifier is trained on* $\mathscr{X} = \{(\boldsymbol{x}_1, y_1), (\boldsymbol{x}_2, y_2), \ldots, (\boldsymbol{x}_N, y_N)\}$ *and outputs the hypothesis* $f(\boldsymbol{x}_i)$*, for each* $i = 1, \ldots, N$*. In addition, with some small probability the classifier's training error is slightly below the one of a random guesser. Since the expected training error of a random guessing classifier is* $1/2$*, one can say that if*

$$\varepsilon = \frac{\sum_{i=1}^{N} \mathbb{1}_{f(\boldsymbol{x}_i) \neq y_i}}{N} \leq \frac{1}{2} - \gamma \tag{2}$$

is true for every real γ *such that* $0 < \gamma \leq \frac{1}{2}$*, then* $f(\boldsymbol{x}_i)$ *represents a* ***weak*** *hypothesis , while the classifier is referred to as* ***weak learner****.*

Regression Stump Weak Learner

A regressive classification model possessing the characteristics of a weak learner is the *regression stump*. It learns an optimal separating line, not a hyperplane, by a single dimension of the input data and predicts the output more accurately than a random guess of the class label. It differs from a single-node decision tree (decision stump) by the error function being minimized by the weak learning algorithm. The regression stump, the function f in line 5 of Algorithm 1, is defined as

$$f_{\mathscr{X}}(x|\tau) = a\mathbb{1}_{x>\tau} + b, \quad a, b \in \mathbb{R}, \tag{3}$$

where $\tau \in \mathbb{R}$ is a threshold value, and a and b are unknown real parameters optimized for any specific value of τ, while f is itself fit using the training data $\mathscr{X}$. The $\mathbb{1} \in \{0,1\}$ is an occurrence indicator for a random event A (the outputs are self-explanatory). For brevity, we leave τ out and use the notation $f_{\mathscr{X}}(x)$. The boosting round $t \in [1,T]$ is omitted because it is clear from context in these definitions. Adapting Equation (3) to our case of classifying an instance $\boldsymbol{x}$, we get

$$f_{\mathscr{X}}(\boldsymbol{x}) = a\mathbb{1}_{x^{(k)}>\tau} + b, \quad a, b \in \mathbb{R}, k \in [1,d], \tag{4}$$

where the k-th dimension coordinate $x^{(k)}$ of $\boldsymbol{x}$ is compared to a threshold value τ.

To train a regression stump it is necessary to search for the threshold τ that yields the smallest possible WSSE ε_t. Let there be given a training dataset $\mathscr{X} \in \mathbb{R}^{N\times d}$ that contains N d-dimensional vectors (instances). The domain of the threshold τ is an unordered set $\mathscr{T} \subset \{x_1^{(k)}, x_2^{(k)}, \ldots, x_N^{(k)}\}$ of d sorted lists $\ell_1, \ldots, \ell_d$, each containing $N-1$ elements in ascending order

$$\ell_k = \left(x_{\alpha(1)}^{(k)}, x_{\alpha(2)}^{(k)}, \ldots, x_{\alpha(N-1)}^{(k)}\right), \quad x_{\alpha(1)}^{(k)} \le x_{\alpha(2)}^{(k)} \le \ldots \le x_{\alpha(N-1)}^{(k)}, k = 1, \ldots, d,$$

where $\alpha : \mathbb{N} \to \mathbb{N}$ is the sort map over the set of all integers, and

$$\mathscr{T} = \{\ell_1, \ldots, \ell_d\}.$$

The largest element by each dimension of $\mathscr{X}$ is deliberately removed in order to avoid the special case of a trivial regression stump that always predicts only one class.

For each $\tau \in \mathscr{T}$ it is necessary to optimize the values of regression coefficients a and b, i.e the optimal $\hat{a}_\tau$ and $\hat{b}_\tau$ are chosen such that

$$(\hat{a}_\tau, \hat{b}_\tau) = \arg\min_{a,b} \sum_{i=1}^{N} w_i \left(y_i - (a\mathbb{1}_{x_i^{(k)}>\tau} + b)\right)^2.$$

This equation is solved by setting the two partial derivatives with respect to a and b to zero. Therefore, the set of solutions $\mathscr{S}$ of the obtained non-homogeneous system is

$$\begin{aligned}\mathscr{S} = \{(a,b) \,|\, &\frac{\partial}{\partial a} \sum_{i=1}^{N} w_i \left(y_i - (a\mathbb{1}_{x_i^{(k)}>\tau} + b)\right)^2 = 0 \wedge \\ &\frac{\partial}{\partial b} \sum_{i=1}^{N} w_i \left(y_i - (a\mathbb{1}_{x_i^{(k)}>\tau} + b)\right)^2 = 0\}.\end{aligned} \tag{5}$$

Furthermore, $|\mathscr{S}| = 1$, i.e., there exists a unique solution to the system, such that $\mathscr{S} = \{(\hat{a}_\tau, \hat{b}_\tau)\}$. Although it involves standard linear algebra theory, we provide a brief theorem for completeness, conciseness and notion of the reader. We use vector notation, where $\boldsymbol{w} = [w_1, w_2, \ldots, w_N]^T$ represents the weights, whilst $\mathbb{1}_{k,\tau} = \left[\mathbb{1}_{x_1^{(k)}>\tau}, \mathbb{2}_{x_i^{(k)}>\tau}, \ldots, \mathbb{N}_{x_i^{(k)}>\tau}\right]^T$ is a vector of indicators and $\boldsymbol{y} = [y_1, y_2, \ldots, y_N]^T$. Additionally, $\langle \cdot, \cdot \rangle$ denotes the *dot (scalar) product* of two vectors.

Supplementary Theorem S1 (Uniqueness of the optimal $(\hat{a}_\tau, \hat{b}_\tau)$ for any τ)**.** *For any threshold $\tau \in \mathscr{T}$, the optimal regression stump coefficients $\hat{a}_\tau$ and $\hat{b}_\tau$ are unique.*

The solutions $\hat{a}_\tau$ and $\hat{b}_\tau$ can be easily derived by algebraic operations and are given by

$$\begin{aligned}\hat{a}_\tau &= \frac{\langle \boldsymbol{w}, \boldsymbol{y}, \mathbb{1}_{k,\tau} \rangle \|\boldsymbol{w}\|_1 - \langle \boldsymbol{w}, \mathbb{1}_{k,\tau} \rangle \langle \boldsymbol{w}, \boldsymbol{y} \rangle}{\langle \boldsymbol{w}, \mathbb{1}_{k,\tau} \rangle \|\boldsymbol{w}\|_1 - \langle \boldsymbol{w}, \mathbb{1}_{k,\tau} \rangle \langle \boldsymbol{w}, \mathbb{1}_{k,\tau} \rangle}, \\ \hat{b}_\tau &= \frac{\langle \boldsymbol{w}, \boldsymbol{y} \rangle - \langle \boldsymbol{w}, \boldsymbol{y}, \mathbb{1}_{k,\tau} \rangle}{\|\boldsymbol{w}\|_1 - \langle \boldsymbol{w}, \mathbb{1}_{k,\tau} \rangle}.\end{aligned} \tag{6}$$

Finally, the optimal regression stump threshold τ^* by any dimension k of $\mathscr{X}$, accompanied by its corresponding optimal regression coefficients $\hat{a}_{\tau^*}$ and $\hat{b}_{\tau^*}$, is chosen such that it minimizes ε_t, or

$$\tau^* = \arg\min_{\tau} \sum_{i=1}^{N} w_i \left(y_i^{(t)} - (\hat{a}_\tau \mathbb{1}_{x_i^{(k)} > \tau} + \hat{b}_\tau) \right)^2, \quad k \in [1, d].$$

The general regression stump algorithm is outlined in Algorithm 2.

Algorithm 2 Regression Stump Algorithm

```
procedure REGRESSION STUMP( 𝒯, weights w, class labels y )
    Let there be N training samples z_1 = (x_1, y_1), z_2 = (x_2, y_2), ..., z_N = (𝒳_N, y_N)
    Initialize the optimal τ* = ∞, â_τ* = ∞, b̂_τ* = ∞, ε_τ* = ∞
    for k = 1, 2, ..., d do
        for i = 1, 2, ..., N − 1 do
            Select a threshold τ ← ℓ_k[i], ℓ_k ∈ 𝒯
            Compute the indicator vector ⟨w, 𝟙_{k,τ}⟩
            Use Equation (6) to compute the optimal regression coefficients â_τ and b̂_τ
            Compute ε_τ ← Σ_{m=1}^{N} w_m (y_m − (â_τ 𝟙_{x_m^(k) > τ} + b̂_τ))^2.
            if ε_τ < ε_τ* then update
                τ* ← τ = ℓ_k[i]
                â_τ* ← â_τ
                b̂_τ* ← b̂_τ
                k* ← k
                ε_τ* ← ε_τ
            end if
        end for
    end for
    Output the final regression stump classifier f_𝒳(x) = â_τ* 𝟙_{x^(k*) > τ*} + b̂_τ*
end procedure
```

S1.3 Collaboration

In this section we present two margin-based collaborative approaches for bagging of boosting ensembles: Weak-Learner Collaboration (W-CLB) and Strong-Learner Collaboration (S-CLB). The realization of collaboration between individual ensembles is done through information exchange, i.e.,exchange of one or more instances. Both approaches aim to reduce the upper bounds on the generalization error of a subbagged boosting model composed of S Gentle Boost ensembles, but the key difference is the stage at which they occur; W-CLB is injected into the training stage of all Gentle Boost ensembles, while S-CLB operates only on prediction-ready ensembles, i.e., in contrast, the latter occurs just after all S ensembles have been fully trained. Not only do they demonstrate a defiance of overfitting, but also deliver an earnest reduction of the generalization error rate.

S1.3.1 W-CLB

We define W-CLB as a two-phase “data reorganizing process”, where we call the phases Pruning Phase (Phase I) and Expansion Phase (Phase II). Both Phases I and II are consecutively, or more precisely, interchangeably repeated at most n_{exc} times, for which an iterator $\tau = 0, \ldots, n_{exc} - 1$ is introduced in order to describe the process, as provided below.

Pruning (Phase I).
The first step of the W-CLB probe injection at an arbitrary boosting round t involves margin pruning. It consists of sorting the instances within each of the training subsets $\mathscr{X}^{(1)}, \ldots, \mathscr{X}^{(S)}$ (each of size ηN) with respect to their real-valued margins obtained from the regression stumps $f_{t,\mathscr{X}^{(1)}}, \ldots, f_{t,\mathscr{X}^{(S)}}$, respectively. The process yields

$$y_{\alpha(1)} f_{t,\mathscr{X}^{(j)}}(\boldsymbol{x}_{\alpha(1)}) \le y_{\alpha(2)} f_{t,\mathscr{X}^{(j)}}(\boldsymbol{x}_{\alpha(2)}) \le \cdots \le y_{\alpha(\eta N)} f_{t,\mathscr{X}^{(j)}}(\boldsymbol{x}_{\alpha(\eta N)}),$$
$$z_{\alpha(i)} \in \mathscr{X}^{(j)}, \; i = 1, \ldots, \eta N, \; j = 1, \ldots, S,$$

where $\alpha(\cdot) : \mathbb{N} \to \mathbb{N}$ is the sorting map defined over the set of all integers.

Second, W-CLB operates on positive margins exclusively, whilst negative margins are omitted from consideration. Therefore, before W-CLB resumes further, a filter is applied to remove the negative margins from the sorted list above, resulting in a sub-list

$$y_{\alpha(p)}f_{t,\mathscr{X}^{(j)}}(\boldsymbol{x}_{\alpha(p)}) \leq y_{\alpha(p+1)}f_{t,\mathscr{X}^{(j)}}(\boldsymbol{x}_{\alpha(p+1)}) \leq \cdots \leq y_{\alpha(\eta N)}f_{t,\mathscr{X}^{(j)}}(\boldsymbol{x}_{\alpha(\eta N)}), \quad p \in [1, \eta N],$$

where $y_{\alpha(p)}f_{t,\mathscr{X}^{(j)}}(\boldsymbol{x}_{\alpha(p)})$ is the smallest non-negative margin, and $\forall i < p, y_{\alpha(i)}f_{t,\mathscr{X}^{(j)}}(\boldsymbol{x}_{\alpha(i)}) < 0$. For brevity, we artificially set $\forall i < p, y_{\alpha(i)}f_{t,\mathscr{X}^{(j)}}(\boldsymbol{x}_{\alpha(i)}) = \infty$.

Expansion (Phase II).
Phase II follows Phase I directly. First and foremost, we ensure that all instance weights within each subset remain unchanged and in their original order and their original assignment to specific instance slots within that subset (we consider an ordered set of instances). At iteration $\tau = 0$, we create a List of Removed Instances (LOR), containing S slots for positions of removed instances corresponding to each subset,

$$\mathrm{LOR}^{\tau}[j] = \alpha(p+\tau).$$

When an instance $z_{\alpha(p+\tau)} \in \mathscr{X}^{(j)}$ at position $p+\tau$ is replaced by another one, we keep the original weight at position $p+\tau$ from $\mathscr{X}$, or simply, the new instance gets the original weight of the replaced one.

Next, after obtaining LOR, a search procedure is initiated to scan the rest of the subsets $\mathscr{X}^{(k)}, k \neq j$. As soon as the search procedure comes across an instance $z = (\boldsymbol{x}, y) \in \mathscr{X}^{(k)}$ for any $k \neq j$, such that $yf_{t,\mathscr{X}^{(j)}}(\boldsymbol{x}) > y_{\mathrm{LOR}[j]^{\tau}}f_{t,\mathscr{X}^{(j)}}(\boldsymbol{x}_{\mathrm{LOR}[j]^{\tau}})$, it terminates. Finally, the new instance z is copied from $\mathscr{X}^{(k)}$ to $\mathscr{X}^{(j)}$ at position $\mathrm{LOR}[j]^{\tau}$, and τ is incremented to $\tau+1$.

Phase I and Phase II are defined for $j \in [1, S]$. Phase I, as well as Phase II, are individually repeated for each $j = 1, \ldots, S$. Afterwards, Phase I is initiated again, when τ increments by 1 and re-initiation is performed until $\tau < n_{exc}$ or until a case when none of the subsets substantiates the conditions for collaborative instance exchange. After W-CLB fully finishes, the Subbagged Gentle Boost algorithm proceeds normally by updating the weights within each subset.

The Algorithm 3 provides the steps of W-CLB for an arbitrary round t. We write $F_{\mathscr{X}^{(j)}}$ to denote the j-th Gentle Boost ensemble being trained on $\mathscr{X}^{(j)}$, hence $F^{(j)} \equiv F_{\mathscr{X}^{(j)}}$. This applies to $f^{(j)}$ as well, for any $j \in [1, S]$. W-CLB selects at most n_{exc} instances from each training subset and replaces them by counterparts that display greater margins at the source weak learner. W-CLB is injected into Subbagged Gentle Boost, resting on a parameter p_c – the probability to run W-CLB at each iteration. For simplicity, this probability is uniformly spread on $[1, T]$, such that W-CLB is performed on every $1/p_c$ rounds of boosting. In Algorithm 3, instance exchange is performed in an iterative manner, i.e., a single instance from each subset at a time. If the swap fails at line 14, then W-CLB examines the next smallest margin in each subset, and so on. The variable *successes* keeps track of the number of successful swaps.

Supplementary Definition S2 (Further-trained classifiers)**.** *Let $\mathscr{X} \in \mathscr{Z}^N, N > 1$ be a training set over which W-CLB is injected at round t of boosting, yielding $\mathscr{X}' \in \mathscr{Z}^N$. Assume that f is a regression stump and F is a Gentle Boost ensemble. Then, a regression stump (respectively a Gentle Boost ensemble) trained according to Algorithm 4 is called a further-trained regression stump, denoted $f^h_{t,\mathscr{X}'}$ and*

$$F^h_{t,\mathscr{X}'}(\boldsymbol{x}) = f^h_{t,\mathscr{X}'}(\boldsymbol{x}) + \sum_{s=1}^{t-1} f_{s,\mathscr{X}}(\boldsymbol{x}), \quad z = (\boldsymbol{x}, y) \in \mathscr{Z},$$

and at iteration $t+1$ there are ordinarily trained $f_{t+1,\mathscr{X}'}$ and $F_{t+1,\mathscr{X}'}$.

Margins. We replicate an existing definition of the margin of an instance $\boldsymbol{x}$ with respect to a classifier f. Since the regression stump outputs real values, or more precisely $f \in [-1, 1]$, one can think of the margin as the distance of $\boldsymbol{x}$ to the decision boundary represented by f. The margin of a misclassified instance is negative because $sign[f(\boldsymbol{x})] \neq y$, and positive otherwise.

Supplementary Definition S3. *(Margins) Let $f \in \mathbb{R}$ be the real-valued outcome of a classification algorithm trained on some $\mathscr{X} \in \mathscr{Z}^N$. Then the margin of $z = (\boldsymbol{x}, y)$ with respect to the decision boundary f is defined as*

$$yf(\boldsymbol{x}), \quad y \in \{-1, 1\}, -1 \leq yf(\boldsymbol{x}) \leq 1.$$

The magnitude of the margin represents the confidence of the decision, while its sign indicates its correctness. In Gentle Boost, the output of the real-valued weak learning algorithm (regression stump) falls between -1 and 1, while the output of the ensemble itself falls between $-T$ and T.

Algorithm 3 Weak-Learner Collaboration (W-CLB)

procedure W_CLB($n_{exc}, f_{t,\mathscr{X}^{(1)}}, \ldots, f_{t,\mathscr{X}^{(S)}}, \boldsymbol{w}_t^{(1)}, \ldots, \boldsymbol{w}_t^{(S)}$)
  Let t be the current round in all S Gentle Boost ensembles
  Ensure: $\boldsymbol{w}_t^{(1)}, \ldots, \boldsymbol{w}_t^{(S)}$ remain unchanged throughout W-CLB
  Save the original training subsets $\mathscr{X}^{(1)}, \ldots, \mathscr{X}^{(S)}$
  $\mathscr{X}^{(j)\prime} \leftarrow \mathscr{X}^{(j)}$, $j = 1, \ldots, S$
  for 1 **to** n_{exc} **do** ▷ *Swapping* loop
    LOR $\leftarrow [\,]_S$ ▷ list of S positions of potentially removed instances
    for $j = 1, \ldots, S$ **do**
      $successes \leftarrow 0$
      $i_{remove_j} = \underset{i: y_i f_{t,\mathscr{X}^{(j)}}(\boldsymbol{x}_i) > 0}{\arg\min} \; y_i f_{t,\mathscr{X}^{(j)}}(\boldsymbol{x}_i), \quad (\boldsymbol{x}_i, y_i) = z_i \in \mathscr{X}^{(j)}$
      $\text{LOR}[j] \leftarrow i_{remove_j}$
      for $k = 1, \ldots, S$, $k \neq j$ **do** ▷ *Search* loop
        for $z \in \mathscr{X}^{(k)}$ **do**
          if $y f_{t,\mathscr{X}^{(j)}}(\boldsymbol{x}) > y_{\text{LOR}[j]} f_{t,\mathscr{X}^{(j)}}(\boldsymbol{x}_{\text{LOR}[j]})$ **then**
            $\mathscr{X}^{(j)\prime} \leftarrow \mathscr{X}^{(j)\prime} \setminus \{z_{\text{LOR}[j]}\} \cup \{z\}$, $z_{\text{LOR}[j]} \in \mathscr{X}^{(j)}$, $z \in \mathscr{X}^{(k)}$
            $successes \leftarrow successes + 1$
            break *Search* loop
          end if
        end for
      end for
      $y_{\text{LOR}[j]} f_{t,\mathscr{X}^{(j)}}(\boldsymbol{x}_{\text{LOR}[j]}) \leftarrow \infty$ ▷ simulate Phase II, i.e., Expansion Phase
    end for
    if $successes = 0$ **then**
      break *Swapping* loop
    end if
  end for
  for $j = 1, \ldots, S$ **do**
    $f_{t,\mathscr{X}^{(j)\prime}} = \text{TrainFurther}(f_{t,\mathscr{X}^{(j)}}, \mathscr{X}^{(j)}, \mathscr{X}^{(j)\prime})$
    $\mathscr{X}^{(j)} \leftarrow \mathscr{X}^{(j)\prime}$
  end for
end procedure

Algorithm 4 Further-Training

procedure FURTHERTRAINING($f_{t,\mathscr{X}}$, $\mathscr{X}$, $\mathscr{X}'$)
  $\varepsilon^{prev} \leftarrow \sum_{m=1}^{|\mathscr{X}'|} w_{mt} \left(1 - y_m f_{t,\mathscr{X}}\right)^2, \quad z_m \in \mathscr{X}'$
  Initialize the optimal $\tau^* = \infty, \hat{a}_{\tau^*} = \infty, \hat{b}_{\tau^*} = \infty, \varepsilon_{\tau^*} = \infty$
  $\mathscr{T} \leftarrow SortAttributeValues(\mathscr{X}' \setminus \mathscr{X})$
  for $k = 1, 2, \ldots, d$ **do**
    for $i = 1, 2, \ldots, |\mathscr{X}' \setminus \mathscr{X}| - 1$ **do**
      Select a threshold $\tau \leftarrow \ell_k[i]$, $\ell_k \in \mathscr{T}$
      Use Equation (6) to compute the optimal regression coefficients $\hat{a}_\tau, \hat{b}_\tau$
      Compute $\varepsilon_t \leftarrow \sum_{m=1}^{|\mathscr{X}'|} w_{mt} \left(y_m - (\hat{a}_\tau \mathbb{1}_{x_m^{(k)} > \tau} + \hat{b}_\tau)\right)^2, \quad z_m \in \mathscr{X}'$
      if $\varepsilon_\tau < \varepsilon_{\tau^*}$ **then** update **then**
        $\tau* \leftarrow \tau = \ell_k[i]$
        $\hat{a}_{\tau^*} \leftarrow \hat{a}_\tau$
        $\hat{b}_{\tau^*} \leftarrow \hat{b}_\tau$
        $k^* = k$
        $\varepsilon_{\tau^*} \leftarrow \varepsilon_\tau$
      end if
    end for
  end for
  if $\varepsilon_{\tau^*} < \varepsilon^{prev}$ **then**
    Construct $f_{t,\mathscr{X}'}^h$ using the new optimal $\tau^*, \hat{a}_{\tau^*}, \hat{b}_{\tau^*}, \hat{b}_{\tau^*}, k^*$
  end if
  Return $f_{t,\mathscr{X}'}^h$
end procedure

The relationship of W-CLB to margin theory. From SVM theory, it is well known that generalization performance is closely related to the increase of margins. The margin of a classifier is defined as the width of the belt region around the decision boundary enclosed by the instances on each side that are closest to the boundary[S3], that is, the two instances having the two smallest margin magnitudes. Moreover, according to Breiman's reasoning, larger minimum margins would intuitively imply lower generalization error[S4]. Therefore, it becomes clear that replacing the instances that define the margin belt by ones that eventually increase it implies lower generalization error. This effect can be thought of as *margin relaxation*, since the new replacements "relax" a tight margin belt around the optimal decision boundary in terms of WSSE. Margin relaxation is often referred to as *minimum margin maximization* since it increases the smallest margin magnitudes within the training data.

An immediate consequence of margin relaxation is enabling re-adaptation of the decision boundary represented by f. Therefore, f, albeit not necessarily, generalizes potentially better after margin relaxation, and ameliorates the risk of overfitting the training data. Geometrically, relaxation enables translation or rotation of the decision boundary. The magnitude $|yf(\boldsymbol{x})|$ of an instance margin represents the confidence of the prediction.

Algorithm 3 depicts two major deeds that are to a certain extent counterintuitive to margin theory, as well as common knowledge and sense.

- W-CLB focuses on and penalizes correctly classified instances. More precisely, W-CLB does not perform classical margin relaxation since it replaces the most uncertain correctly predicted samples. These instances do not necessarily define the margin belt around the optimal f_t, hence W-CLB may fail to widen the belt itself, especially when f_t is cluttered by more than n_{exc} mispredicted samples with a negligible confidence. In that case, the margin belt remains unchanged and W-CLB has a heavily "diminished" effect of relaxation. W-CLB is thus contrasting to improving the generalization error based on minimal margins.

- On the other hand, W-CLB operates contrarily to common knowledge and facts in margin theory because it penalizes correct predictions. Common sense implies that it is best to replace the instance that has the smallest negative margin (that is, the most confident wrong prediction) by an instance that has a much greater, positive margin. In the next section, we stress this phenomenon and provide several reasons to justify the actual W-CLB approach.

Although seemingly counterintuitive, Section S2 makes it clear that highly confident correct predictions can be leveraged to fine-tune an optimal weak decision boundary, and most importantly, improve the overall algorithmic stability of Subbagged Gentle Boost.

S1.3.2 S-CLB

The following part of the text provides information about the concept of S-CLB. This collaboration procedure is conducted through $\mathscr{T}$ consecutive iterations. At the τ-th iteration of S-CLB, the j-th Gentle Boost ensemble initiates a collaboration procedure referring to its k-th predecessor within the ensemble sequence. By doing so, they form a collaboration pair (j,k). This collaboration pair goes through three steps described in detail bellow and also presented in Algorithm 5.

Collaboration criterion satisfaction (Step I).
Let $F^{(\tau)}_{\mathscr{X}^{(j,\tau)}}$ and $F^{(\tau)}_{\mathscr{X}^{(k,\tau)}}$ represent the outcomes of the j-th and the k-th Gentle Boost ensemble, respectively, where $\mathscr{X}^{(j,\tau)}$ and $\mathscr{X}^{(k,\tau)}$ denote the corresponding datasets used for their training at the τ-th iteration. First of all, the instance-label pairs contained in the j-th ensemble's training set which are not used to train its predecessor are selected, thus forming the relative complement $\mathscr{X}^{(j\backslash k,\tau)} = \mathscr{X}^{(j,\tau)} \backslash \mathscr{X}^{(k,\tau)}$. This is done the other way around as well. Furthermore, the margins of all instance-label pairs in $\mathscr{X}^{(j\backslash k,\tau)}$ with respect to $F^{(\tau)}_{\mathscr{X}^{(j,\tau)}}$, as well as the ones in $\mathscr{X}^{(k\backslash j,\tau)}$ with respect to $F^{(\tau)}_{\mathscr{X}^{(k,\tau)}}$, are sorted in an ascending order. So, if $M^{(\tau)}_j(z)$ denotes the margin of a given pair $z = (\boldsymbol{x}, y)$ with respect to the j-th Gentle Boost ensemble at the τ-th iteration, i.e., $M^{(\tau)}_j(z) = yF^{(\tau)}_{\mathscr{X}^{(j,\tau)}}(\boldsymbol{x}), \forall z \in \mathscr{Z}$, then the corresponding sorted margin sequence regarding this ensemble is the following

$$M^{(\tau)}_j(z_{u_1}) \leq M^{(\tau)}_j(z_{u_2}) \leq \cdots \leq M^{(\tau)}_j(z_{u_{N_{j\backslash k,\tau}}}),$$

where $u_p \neq u_q$ and $u_p, u_q \in [1,N]$, for each $p \neq q$ $(p,q = 1,\dots,N_{j\backslash k,\tau})$. Analogously, in the case of the k-th Gentle Boost ensemble we have

$$M^{(\tau)}_k(z_{v_1}) \leq M^{(\tau)}_k(z_{v_2}) \leq \cdots \leq M^{(\tau)}_k(z_{v_{N_{k\backslash j,\tau}}}),$$

where $v_p \neq v_q$ and $v_p, v_q \in [1,N]$, for each $p \neq q$ $(p,q = 1,\dots,N_{k\backslash j,\tau})$. Note that $z_{u_1}, z_{u_2}, \dots,$ $z_{u_{N_{j\backslash k,\tau}}}$ and $z_{v_1}, z_{v_2}, \dots, z_{v_{N_{k\backslash j,\tau}}}$ essentially represent the elements of $\mathscr{X}^{(j\backslash k,\tau)}$ and $\mathscr{X}^{(k\backslash j,\tau)}$, respectively. Now, these two base ensembles may proceed to collaborate only if both sets $\mathscr{X}^{(j\backslash k,\tau)}$ and $\mathscr{X}^{(k\backslash j,\tau)}$ are not empty. Otherwise, there is no training information that can be exchanged between these two ensembles in the next step. On the other hand, if the number of instance-label pairs learned by just one of the ensembles is larger then the pre-set parameter n_{exc}, then n_{exc} should be chosen as the maximal number of instances allowed to be exchanged throughout the first step at the τ-th iteration. This number is denoted by $n^{(\tau)}_{exc}$. Accordingly, the collaboration procedure regarding the pair (j,k) may continue only if the following is satisfied

$$n^{(\tau)}_{exc} = \min\{n_{exc}, |\mathscr{X}^{(j\backslash k,\tau)}|, |\mathscr{X}^{(k\backslash j,\tau)}|\} > 0.$$

Probe exchange of training information (Step II).
As mentioned previously, a convenient method of exchanging training information between two base Gentle Boost ensembles would be one that considers an exchange of training instances. In our case, this method is applied such that the exchange of training instances essentially represents an instance swapping. By "swapping" it is meant that each time an instance is removed from a given training subset and added to another one, it must be replaced by an instance drawn from the latter subset. This provides consistency in terms of the fact that the cardinality of each training subset will always remain the same, while its contents may vary. As to the instance swapping between the j-th and the k-th Gentle Boost ensemble at the τ-th iteration, some or all of the top $n^{(\tau)}_{exc}$ instance-label pairs from $\mathscr{X}^{(j\backslash k,\tau)}$ whose margins with respect to $F^{(\tau)}_{\mathscr{X}^{(j,\tau)}}$ have the smallest values are swapped with the corresponding ones from $\mathscr{X}^{(k\backslash j,\tau)}$ having the smallest margins with respect to $F^{(\tau)}_{\mathscr{X}^{(k,\tau)}}$. Thus, the indices of these instance from both $\mathscr{X}^{(j\backslash k,\tau)}$ and $\mathscr{X}^{(k\backslash j,\tau)}$, are separated into different sets $\mathscr{I}^{(j\backslash k,\tau)} = \{u_1,\dots,u_{n^{(\tau)}_{exc}}\}$ and $\mathscr{I}^{(k\backslash j,\tau)} = \{v_1,\dots,v_{n^{(\tau)}_{exc}}\}$. The former contains the indices of all instances which may potentially be swapped with those instances whose indices are contained in latter. But, these instances are swapped according to a certain swapping order. A potential swapping order is defined simply as a set of pairs such that a given pair (u,v) within the set represents the swap between $z_u \in \mathscr{X}^{(j\backslash k,\tau)}$ and $z_v \in \mathscr{X}^{(k\backslash j,\tau)}$, where $u \in \mathscr{I}^{(j\backslash k,\tau)}$ and $v \in \mathscr{I}^{(k\backslash j,\tau)}$. Accordingly, the set of all potential swapping orders is the following

$$\mathscr{S}^{(j,k,\tau)} = \left\{ \mathscr{S}^{(j,k,\tau)}_{n,p_j,p_k} \right\}_{n=1}^{n^{(\tau)}_{exc}} {}_{p_j=1}^{\binom{n^{(\tau)}_{exc}}{n}} {}_{p_k=1}^{\binom{n^{(\tau)}_{exc}}{n}}.$$

In essence, if $\mathscr{P}(A)$ denotes the relative complement of the power set of a given set A with respect to $\varnothing$ and $g : \mathscr{P}(\mathscr{I}^{(j\setminus k,\tau)})_{n,p_j} \to \mathscr{P}(\mathscr{I}^{(k\setminus j,\tau)})_{n,p_k}$ is a bijective function between the elements of the p_j-th subset of $\binom{\mathscr{P}(\mathscr{I}^{(j\setminus k,\tau)})}{n}$ and the p_k-th subset of $\binom{\mathscr{P}(\mathscr{I}^{(k\setminus j,\tau)})}{n}$, then each constituent subset of $\mathscr{S}^{(j,k,\tau)}$, i.e., each potential swapping order can be defines as

$$\mathscr{S}^{(j,k,\tau)}_{n,p_j,p_k} = \{(u_{n,p_j,p_k,1}, v_{n,p_j,p_k,1}), \ldots, (u_{n,p_j,p_k,n}, v_{n,p_j,p_k,n})\},$$

where $(u_{n,p_j,p_k,1}, v_{n,p_j,p_k,1}), \ldots, (u_{n,p_j,p_k,n}, v_{n,p_j,p_k,n})$ are all unique such that $v_{n,p_j,p_k,b} = g(u_{n,p_j,p_k,b})$, for each $u_{n,p_j,p_k,b} \in \mathscr{P}(\mathscr{I}^{(j\setminus k,\tau)})_{n,p_j}$, $v_{n,p_j,p_k,b} \in \mathscr{P}(\mathscr{I}^{(k\setminus j,\tau)})_{n,p_k} (n = 1, \ldots, n^{(\tau)}_{exc},\ p_j, p_k = 1, \ldots, \binom{n^{(\tau)}_{exc}}{n}$,
$b = 1, \ldots, n)$. For simplicity of notation, we use $\mathscr{S}^{(j,k,\tau)}_p$ to denote a swapping order and $(u_{p,b}, v_{p,b})$ to denote a swapping pair, i.e.,$\mathscr{S}^{(j,k,\tau)}_p \equiv \mathscr{S}^{(j,k,\tau)}_{n,p_j,p_k}$ and $(u_{p,b}, v_{p,b}) \equiv (u_{n,p_j,p_k,b}, v_{n,p_j,p_k,b})$, $\forall p = 1, \ldots, n^{(\tau)}_{swap} = n^{(\tau)}_{exc}\binom{n^{(\tau)}_{exc}}{n}\binom{n^{(\tau)}_{exc}}{n}, b = 1, \ldots, |\mathscr{S}^{(j,k,\tau)}_p|$. All of these swapping orders are iterated and a probe exchange of instance-label pairs is conducted according to each of them. More specifically, given a potential swapping order $\mathscr{S}^{(j,k,\tau)}_p \in \mathscr{S}^{(j,k,\tau)}$, each of its constituent swapping pairs $(u_{p,b}, v_{p,b})$ $(b = 1, \ldots, |\mathscr{S}^{(j,k,\tau)}_p|)$ is used to obtain the following updates:

$$\mathscr{X}^{(j,\tau)}_p = \left(\mathscr{X}^{(j,\tau)} \setminus \{z_{u_{p,b}}\}\right) \cup z_{v_{p,b}},$$

$$\mathscr{X}^{(k,\tau)}_p = \left(\mathscr{X}^{(k,\tau)} \setminus \{z_{v_{p,b}}\}\right) \cup z_{u_{p,b}},$$

where $\mathscr{X}^{(j,\tau)}_p$ and $\mathscr{X}^{(k,\tau)}_p$ represent the modifications of $\mathscr{X}^{(j,\tau)}$ and $\mathscr{X}^{(k,\tau)}$ after the occurrence of the p-th probe exchange. The procedure is repeated for each $p = 1, \ldots, n^{(\tau)}_{swap}$.

Satisfaction of a criterion for successful information exchange (Step III).
Upon completion of all probe exchanges, the last step at the τ-th iteration evaluates the successfulness of each one of them. It is the most important step in terms of the model's performance improvement which will be discussed later. In other words, the decision regarding the model's state-change is made within this step. Essentially, after each probe exchange, the model's state is modified, but just **temporarily**. To decide whether the model's state will be permanently modified, the results from all possible probe exchanges made within Step II are examined and the successfulness of each is measured. For this purpose, a distance metric is evaluated **before** and **after** each probe exchange between the j-th and the k-th Gentle Boost ensemble. Afterwards, the measured distances are used to compare the initial model at the beginning of the τ-th iteration against its modification caused by the probe exchange conducted according to the p-th swapping order, for each $p = 1, \ldots, n^{(\tau)}_{swap}$. If an optimal swapping order is found in terms of the model's performance, then the model's state is modified accordingly and the τ-th iteration is referred to as state-changing.

To quantify the distances, first, the losses of both $F^{(\tau)}_{\mathscr{X}^{(j,\tau)}}$ and $F^{(\tau)}_{\mathscr{X}^{(k,\tau)}}$ are calculated with respect to each instance z_i within the original training set $\mathscr{X}$. Next, the empirical errors of both ensembles are calculated, before and after the p-th instance exchange. In our case, the empirical error differences of the j-th and the k-th Gentle Boost ensemble are

$$R^{(j,\tau)}_{p,diff} = R^T_{emp}(F^{(\tau)}_{\mathscr{X}^{(j,\tau)}}, \mathscr{X}^{(j,\tau)}) - R^T_{emp}(F^{(\tau)}_{\mathscr{X}^{(j,\tau)}_p}, \mathscr{X}^{(j,\tau)}_p)$$

and

$$R^{(k,\tau)}_{p,diff} = R^T_{emp}(F^{(\tau)}_{\mathscr{X}^{(k,\tau)}}, \mathscr{X}^{(k,\tau)}) - R^T_{emp}(F^{(\tau)}_{\mathscr{X}^{(k,\tau)}_p}, \mathscr{X}^{(k,\tau)}_p),$$

respectively. These two measures are combined in the following error distance measure

$$dist^{(j,k,\tau)}_p = \left\| \left[R^{(j,\tau)}_{p,diff}\ \ R^{(k,\tau)}_{p,diff}\right]^T \right\|_2.$$

Afterwards, the sufficiency of the distance value is examined. This is done by defining an indicator variable with respect to the p-th probe exchange as follows

$$I^{(j,k,\tau)}_p = \mathbb{1}_{R^{(j,\tau)}_{p,diff} \geq 0 \,\wedge\, R^{(k,\tau)}_{p,diff} \geq 0}.$$

Note that the value of the distance measure $dist_p^{(j,k,\tau)}$, as well as the value of the indicator variable $I_p^{(j,k,\tau)}$, are calculated in the case of each probe exchange of instances, i.e., for each $p = 1, \ldots, n_{swap}^{(\tau)}$.

At last, the optimal instance exchange, i.e., the optimal swapping order is the one that maximizes $dist_p^{(j,k,\tau)}$. Therefore, the optimal swapping order $\mathscr{S}_{p^*}^{(j,k,\tau)}$ is determined by obtaining the following optimization

$$
\begin{aligned}
\arg\max_{p} \quad & I_p^{(j,k,\tau)} dist_p^{(j,k,\tau)} \\
\text{s.t.} \quad & p \in \{1, \ldots, n_{swap}^{(\tau)}\}.
\end{aligned}
$$

Accordingly, the training sets' contents regarding both Gentle Boost ensembles are updated using $\mathscr{S}_{p^*}^{(j,k,\tau)}$,

$$
\mathscr{X}^{(j,\tau+1)} = \mathscr{X}_{p^*}^{(j,\tau)}
$$

$$
\mathscr{X}^{(k,\tau+1)} = \mathscr{X}_{p^*}^{(k,\tau)}.
$$

The procedure is repeated at each iteration $\tau = 0, \ldots, \mathscr{T} - 1$, i.e., for all pairs of type (j,k), where $j = 1, \ldots, S$ and $k = j-1, \ldots, 1$. Accordingly, the training process of the Subbagged Gentle Boost model consists of $\mathscr{T} = \sum_{j=2}^{S} j = (S-1)S/2$ iterations overall, while $\mathscr{T}_{sc} \leq \mathscr{T}$ of them are state-changing. Its step-by-step algorithmic description is presented below.

Algorithm 5 Strong-Learner Collaboration (S-CLB)

procedure S_CLB(max number of instances allowed to be exchanged n_{exc}, collaboration pair (j,k), outcomes $F^{(\tau)}_{\mathscr{X}^{(1,\tau)}}, \ldots, F^{(\tau)}_{\mathscr{X}^{(S,\tau)}}$, number of state-changing iterations $\mathscr{T}_{sc}$)

  $\mathscr{X}^{(j\backslash k,\tau)} \leftarrow \mathscr{X}^{(j,\tau)} \backslash \mathscr{X}^{(k,\tau)}$

  $\mathscr{X}^{(k\backslash j,\tau)} \leftarrow \mathscr{X}^{(k,\tau)} \backslash \mathscr{X}^{(j,\tau)}$

  Sort instances with respect to their margins

  $\left[M_j^{(\tau)}(z_{u_1})\ M_j^{(\tau)}(z_{u_2})\ \ldots\ M_j^{(\tau)}(z_{u_{N_{j\backslash k,\tau}}})\right]^T \leftarrow \textbf{Sort}\left(\left[M_j^{(\tau)}(z)\right]_{\forall z \in \mathscr{X}^{(j\backslash k,\tau)}}\right)$

  $\left[M_k^{(\tau)}(z_{v_1})\ M_k^{(\tau)}(z_{v_2})\ \ldots\ \ldots M_k^{(\tau)}(z_{v_{N_{k\backslash j,\tau}}})\right]^T \leftarrow \textbf{Sort}\left(\left[M_k^{(\tau)}(z)\right]_{\forall z \in \mathscr{X}^{(k\backslash j,\tau)}}\right)$

  $n_{exc}^{(\tau)} \leftarrow \min\{n_{exc}, |\mathscr{X}^{(j\backslash k,\tau)}|, |\mathscr{X}^{(k\backslash j,\tau)}|\}$

  if $n_{exc}^{(\tau)} > 0$ **then**

    $\mathscr{I}^{(j\backslash k,\tau)} \leftarrow \{u_1, \ldots, u_{n_{exc}^{(\tau)}}\}$

    $\mathscr{I}^{(k\backslash j,\tau)} \leftarrow \{v_1, \ldots, v_{n_{exc}^{(\tau)}}\}$

    Generate the set of all potential swapping orders $\mathscr{S}^{(j,k,\tau)}$ using $\mathscr{I}^{(j\backslash k,\tau)}$ and $\mathscr{I}^{(k\backslash j,\tau)}$

    for $p = 1, \ldots, n_{swap}^{(\tau)}$ **do**

      Exchange instances between the j-th and the k-th ensemble according to $\mathscr{S}_p^{(j,k,\tau)}$

      for each $(u,v) \in \mathscr{S}_p^{(j,k,\tau)}$ **do**

        $\mathscr{X}_p^{(j,\tau)} \leftarrow \left(\mathscr{X}^{(j,\tau)} \backslash \{z_u\}\right) \cup z_v$

        $\mathscr{X}_p^{(k,\tau)} \leftarrow \left(\mathscr{X}^{(k,\tau)} \backslash \{z_v\}\right) \cup z_u$

      end for

      Calculate the distance $dist_p^{(j,k,\tau)}$

    end for

    Initialize $\mathscr{S}_{p^*}^{(j,k,\tau)} \leftarrow \varnothing$

    $dist_{p^*}^{(j,k,\tau)} \leftarrow \arg\max_p I_p^{(j,k,\tau)} dist_p^{(j,k,\tau)}$ s.t. $p \in \{1, \ldots, n_{swap}^{(\tau)}\}$

    if $dist_{p^*}^{(j,k,\tau)} > 0$ **then** update

      $\mathscr{X}^{(j,\tau+1)} \leftarrow \mathscr{X}_{p^*}^{(j,\tau)}$

      $\mathscr{X}^{(k,\tau+1)} \leftarrow \mathscr{X}_{p^*}^{(k,\tau)}$

      $\mathscr{T}_{sc} \leftarrow \mathscr{T}_{sc} + 1$

    end if

  end if

end procedure

S1.4 Stability

In this part we provide a strong theoretical background for collaboration. The work presented here is based on existing stability theory and upper bounds on the generalization errors of bagging and boosting algorithms.

Notation and Preliminaries

The notation and context in this part is mostly adopted from[S5]. Let the sets $\mathscr{D} \subset \mathbb{R}^d, \mathscr{Y} \subset \mathbb{R}$ be the input and output space, respectively. When dealing with a binary classification problem, $\mathscr{Y}$ is constrained to the values $\{-1, +1\}$. Further, let $\mathscr{Z} = \mathscr{D} \times \mathscr{Y}$ denote a learning space of input-output pairs. We consider the space of training sets $\mathscr{Z}^N$ representing all training sets $\mathscr{X}$ of size N drawn i.i.d. from an unknown distribution D. Therefore,

$$\mathscr{X} = \{z_1 = (\boldsymbol{x}_1, y_1), \dots, z_N = (\boldsymbol{x}_N, y_N)\} \in \mathscr{Z}^N.$$

A learning algorithm A is a function which maps a training (learning) set $\mathscr{X}$ onto a function $A_{\mathscr{X}}$ from $\mathscr{D}$ to $\mathscr{Y}$. It is assumed that the algorithm A is *symmetric* with respect to $\mathscr{X}$, which means that the algorithm does not depend on the order of elements in the training set. Furthermore, it is assumed that all functions are measurable and all sets are countable[S5].

For a given training set $\mathscr{X}$ of size N, two operations are considered for building a modified training set for all $i = 1, \dots, N$ as follows:

- By removing the i-th element

$$\mathscr{X}^{\setminus i} = \{z_1, \dots, z_{i-1}, z_{i+1}, \dots, z_N\}.$$

- By replacing the i-th element by some $z \in \mathscr{Z}$ drawn from D and independent of $\mathscr{X}$

$$\mathscr{X}^{\setminus i \cup z} = \{z_1, \dots, z_{i-1}, z, z_{i+1}, \dots, z_N\}.$$

Unless they are clear from context, the random variables over which probabilities or expectations are taken are specified in the subscript. This way, $\mathbf{P}_{\mathscr{X}}[.]$ and $\mathbf{E}_{\mathscr{X}}[.]$ are taken to denote the probability and expectation with respect to a random draw of the training set $\mathscr{X}$ of size N, according to the distribution D^N[S5].

In order to measure the accuracy of the algorithm A we need a measure of loss (a cost function) of a hypothesis f with respect to an instance $z = (\boldsymbol{x}, y)$. We will denote the loss of a hypothesis $f_{\mathscr{X}}$ (respectively the algorithm $A_{\mathscr{X}}$) by $\ell(f_{\mathscr{X}}, z)$ (or equivalently $\ell(A_{\mathscr{X}}, z)$) and quantify it by a cost function $c : \mathscr{Y} \times \mathscr{Y} \to \mathbb{R}^+$. For brevity, the distribution D of $\mathscr{X}$ is left out, and sometimes, unless it is clear from context, the subscript that denotes the training set will also be left out for an additional simplification of notation. Thus,

$$\ell(f_{\mathscr{X}}, z) = c(f_{\mathscr{X}}, y).$$

Note that $\ell(A_{\mathscr{X}}, z) \equiv \ell(f_{\mathscr{X}}, z) \equiv \ell(f, z)$.

Albeit the leave-one-out error is considered to be an unbiased estimate of the true error of an algorithm, Bousquet and Elisseeff showed in[S5] that their upper bounds on the true, i.e., *generalization* error based on leave-one-out error are strikingly similar to those based on the training (empirical) error. Formally, the generalization error $R(A, \mathscr{X})$ depending on the training set $\mathscr{X}$ for A is defined as

$$R(A, \mathscr{X}) = \mathbf{E}_z [\ell(A_{\mathscr{X}}, z)], \quad z \in \mathscr{Z}.$$

Unfortunately, R cannot be computed since the distribution D is unknown. We thus have to estimate it from the available data $\mathscr{X}$[S5]. As stated previously, there are two widely used estimators for the error $R(A, \mathscr{X})$ - the classical leave-one-out error estimator and the so-called *empirical*, i.e.,*training* error. Since the results in[S5] are strikingly similar for both, in our work we focus on the latter, R_{emp}, where

$$R_{emp}(A, \mathscr{X}) = \frac{1}{N} \sum_{i=1}^{N} \ell(A_{\mathscr{X}}, z_i).$$

Definitions of Stability

There are many ways to quantify or define an algorithm's stability, but for the reader unschooled in statistical/computational learning theory, stability is simply the tolerance, or more precisely, the resistance of an algorithm to small changes in the

training data. A stable algorithm will demonstrate very similar generalization performance when trained on different, unalike training sets. The notion of stability was first introduced (not explicitly[S5]) in 1979 by Devroye and Wagner when they analyzed the error variance of local learning algorithms in Distribution-free inequalities for the deleted and holdout error estimates, and referring to[S5], Kearns and Ron defined it and gave it a name in 1999 in[S6]. We now continue with formal stability definitions and theorems that provide upper bounds on R as the theoretical basis of W-CLB and S-CLB. For each existing theorem or lemma, we specify the original source and theorem or lemma number that the reader can refer to for the complete proof, which is often prolix or complicated to be presented here.

Supplementary Definition S4 (Hypothesis stability[S5])**.** *An algorithm A has hypothesis stability β with respect to the loss function ℓ if the following holds*

$$\forall i \in \{1,\dots,N\}, z \in \mathscr{Z}, \mathbf{E}_{\mathscr{X},z}\left[|\ell(A_{\mathscr{X}},z) - \ell(A_{\mathscr{X}^{\setminus i}},z)|\right] \leq \beta. \tag{7}$$

This is equal to the expected L_1 norm with respect to our unknown distribution D.

Supplementary Definition S5 (Pointwise hypothesis stability[S7])**.** *An algorithm A has pointwise hypothesis stability β with respect to the loss function ℓ if the following holds*

$$\forall i \in \{1,\dots,N\}, z \in \mathscr{Z}, \mathbf{E}_{\mathscr{X}}\left[|\ell(A_{\mathscr{X}},z_i) - \ell(A_{\mathscr{X}^{\setminus i \cup z}},z_i)|\right] \leq \beta. \tag{8}$$

With pointwise hypothesis stability, we take the pointwise average (expectation) of the loss perturbations measured on a single instance z_i instead of averaging over the whole $\mathscr{X}$.

Supplementary Definition S6 (Uniform stability[S7])**.** *An algorithm A has uniform stability β with respect to the loss function ℓ if the following holds*

$$\forall \mathscr{X} \in \mathscr{Z}^N, \forall i \in \{1,\dots,N\}, \|\ell(A_{\mathscr{X}},.) - \ell(A_{\mathscr{X}^{\setminus i}},.)\|_\infty \leq \beta. \tag{9}$$

It is important to note that β is an inversely proportional quantification of stability; as β decreases, stability increases proportionally, and moreover, β is a function of N (sometimes denoted β_N) and the case of interest is when β decreases as $1/N$, i.e., β is proportional to $O(1/N)$[S5].

In some cases, like boosting, the algorithm A works on weighted data, and we thus define an appropriate weighted notion of stability, the so-called L_1-stability, where the input set is modified in terms of the weight distribution, instead of element removal/replacement.

Supplementary Definition S7 (L_1-Stability[S8])**.** *An algorithm A has L_1-stability λ, or is λ-L_1-stable with respect to the loss function ℓ, if for any two distributions p and q on $\mathscr{D}$ the following holds*

$$\forall z \in \mathscr{Z}, \mathbf{E}_{\mathscr{X},z}|\ell(A_{\mathscr{X}\sim p},z) - \ell(A_{\mathscr{X}\sim q},z)| \leq \lambda \|p-q\|, \tag{10}$$

where $\|p-q\| = \sum_i |p_i - q_i|$.

This definition was not originally based on the expectation, but was instead defined in terms of raw absolute differences in Definition 2.11[S8]. Defining it in terms of expectation does not imply any conflicts with the existing theory. Interestingly, the L_1 stability is related to hypothesis stability (respectfully pointwise), which is necessary for future analysis.

Supplementary Lemma S1 (Lemma 2.12[S8])**.** *A learning algorithm has L_1-stability λ if and only if it has pointwise hypothesis stability $2\lambda/N$. Furthermore, if a learning algorithm has L_1-stability λ, it has hypothesis stability $2\lambda/N$.*

Finally, the following part focuses on upper bounds on the error that hold in certain circumstances, i.e., hold with some minimum probability. We thus provide a definition of (β,δ) stability.

Supplementary Definition S8 ((β,δ) Stability[S8])**.** *We say that an algorithm A is (β,δ) stable at $\mathscr{X}$ if*

$$\mathbf{P}_{\mathscr{X}\sim D^N}[A \text{ is } \beta\text{-stable at } \mathscr{X}] \geq 1-\delta. \tag{11}$$

Classification Stability. Bousquet and Elisseeff introduce a modified cost function applicable for real-valued classification algorithms which considers the so-called "soft margins". According to[S5], if an algorithm is a real-valued classification algorithm which returns the function $f_{\mathscr{X}}$, then for any $\gamma > 0$, the modified cost function regarding the prediction of a given pair $z = (\boldsymbol{x}, y)$ is defined as

$$c_\gamma(f_{\mathscr{X}}(\boldsymbol{x}), y) = \begin{cases} 1, & \text{if } yf_{\mathscr{X}}(\boldsymbol{x}) \leq 0 \\ 1 - yf_{\mathscr{X}}(\boldsymbol{x})/\gamma, & \text{if } 0 < yf_{\mathscr{X}}(\boldsymbol{x}) \leq \gamma \\ 0, & otherwise \end{cases}, \tag{12}$$

and the adequate algorithm's loss $\ell_\gamma(f_{\mathscr{X}}, z) = c_\gamma(f_{\mathscr{X}}(\boldsymbol{x}), y)$ is called *classification loss*. This soft-margin-based definition of the loss function is a suitable choice for evaluating the quality of the decisions made by a certain real-valued algorithm for two reasons. First of all, although it can be adapted for use in the case of regression problems, it is intended to be used when dealing with classification ones. Moreover, it is considered to be more "flexible" in terms of its ability to distinguish between reliable and unreliable predictions, rather than focusing only on their accuracy. This is achieved by the function's dependency on the value of γ. In fact, the loss will increase as the value of $f_{\mathscr{X}}$ approaches zero, where its critical closeness to zero is controlled by γ.

Now, with the choice of the loss function being settled, we can define the stability measures associated with it. The first and more general stability measure, based only on a classifier's output, is the classification stability, while the second one takes the classification loss into consideration. The two measures are related such that this relation is dictated by γ. The formal definition of the former, as well as the lemma regarding the connection between both measures are presented in the text that follows.

Supplementary Definition S9 (Classification stability[S5])**.** *Let A be a real-valued classification algorithm which returns the function f. The algorithm A has a classification stability β, if for any instance-label pair $z = (\boldsymbol{x}, y)$ the following holds*

$$\forall \mathscr{X} \in \mathscr{Z}^N, \forall i \in \{1, \ldots, N\}, \|f_{\mathscr{X}}(\boldsymbol{x}) - f_{\mathscr{X}^{\setminus i}}(\boldsymbol{x})\|_\infty \leq \beta. \tag{13}$$

Note that $\mathscr{X}^{\setminus i}$ denotes the training set $\mathscr{X}$ after the removal of its i-th element, i.e., $\mathscr{X}^{\setminus i} = \mathscr{X} \setminus \{z_i\}$, meaning that $f_{\mathscr{X}}(\boldsymbol{x})$ and $f_{\mathscr{X}^{\setminus i}}(\boldsymbol{x})$ represent the outputs of a classifier, trained by means of A on $\mathscr{X}$, with and without learning z_i, respectively.

Supplementary Lemma S2 (Lemma 16[S5])**.** *A real-valued classification algorithm A with classification stability β has a uniform stability $\frac{\beta}{\gamma}$ with respect to the classification loss function ℓ_γ.*

The proof of the lemma presented in[S5] states that c_γ is a $1/\gamma$-Lipschitzian function with respect to its argument representing the classifier's output $f_{\mathscr{X}}(\boldsymbol{x})$ for any instance $\boldsymbol{x}$. Consequently, ℓ_γ is also $1/\gamma$-Lipschitzian. Thus, for all i, all training sets $\mathscr{X}$, and all $z = (\boldsymbol{x}, y)$,

$$|\ell_\gamma(f_{\mathscr{X}}, z) - \ell_\gamma(f_{\mathscr{X}^{\setminus i}}, z)| = |c_\gamma(f_{\mathscr{X}}(\boldsymbol{x}), y) - c_\gamma(f_{\mathscr{X}^{\setminus i}}(\boldsymbol{x}), y)| \leq \frac{1}{\gamma}|f_{\mathscr{X}}(\boldsymbol{x}) - f_{\mathscr{X}^{\setminus i}}(\boldsymbol{x})| \leq \frac{\beta}{\gamma}. \tag{14}$$

We can thus see that γ plays the role of controlling the connection between the classification and uniform stability of A. More precisely, it regulates their ratio.

Upper Bounds on the Generalization Error

This part is a brief overview of existing proved upper bounds of the generalization error R of learning algorithms. These bounds are based on VC dimensions and were introduced by Bartlett et al. in[S9]. Later Bousquet and Elisseeff[S5;S7] presented upper bounds based on stability, applicable to a large class of learning algorithms, including real-valued classification algorithms and penalizing algorithms, also known as regularization algorithms. Our work involves stability-based upper bounds of R. Starting from existing AdaBoost bounds given in[S8], we extend stability notions to Gentle Boost and Subbagged Gentle Boost. The following theorem that applies to every majority hypothesis F regardless of how it is computed is from[S9] (it is provided for completeness):

Supplementary Theorem S2 (Theorem 1[S9])**.** *Let $\mathscr{X}$ be a sample of N examples chosen independently at random according to D. Assume that the base (weak) hypothesis space $\mathscr{H}$ is finite, and let $\delta > 0$. Then with probability at least $1 - \delta$ over the random choice of the training set $\mathscr{X}$, every weighted average function F satisfies the following bound for all $\theta > 0$*

$$\mathbf{P}_D\left[yF(x) \leq 0\right] \leq \mathbf{P}_{\mathscr{X}}\left[yF(x) \leq \theta\right] + O\left(\frac{1}{\sqrt{N}}\left(\frac{\ln N \ln |\mathscr{H}|}{\theta^2} + \ln(1/\delta)\right)^{1/2}\right). \tag{15}$$

More generally, for finite or infinite $\mathscr{H}$ with VC dimension d, the following bound holds as well:

$$\mathbf{P}_D\left[yF(x) \leq 0\right] \leq \mathbf{P}_{\mathscr{X}}\left[yF(x) \leq \theta\right] + O\left(\frac{1}{\sqrt{N}}\left(\frac{d\ln^2(N/d)}{\theta^2} + \ln(1/\delta)\right)^{1/2}\right). \tag{16}$$

Note that in the theorem above, $\mathbf{P}_D\left[yF(x) \leq 0\right] = R$ for AdaBoost, where the loss function ℓ is taken to be the Heaviside function of $-yF(x)$, the raw classification loss.

Upper Bounds of Deterministic Algorithms

Switching back to stability theory, there are two major classes of upper bounds on R that are independent of the VC dimension of the base hypotheses - regression-based and classification-based. As mentioned previously, for the latter, the authors in[S5] introduce a modified loss function $\ell_\gamma(A_{\mathscr{X}}, z)$ over $\mathscr{Z}$ for a real-valued classification algorithm A. This is a notion of the so-called *soft margins* which will be later used to provide the theoretical background of S-CLB. On the other hand, in the case of W-CLB, we are solely interested in sign$[f(x)]$, and we thus apply the regression case.

Supplementary Theorem S3 (Theorem 12[S5])**.** *Let A be an algorithm with uniform (resp. hypothesis and pointwise hypothesis) stability β with respect to a loss function ℓ such that $0 \leq \ell(A_{\mathscr{X}}, z) \leq M$, for all $z \in \mathscr{Z}$ and all sets $\mathscr{X}$. Then, for any $N \geq 1$, and any $\delta \in (0,1)$, the following bounds hold with probability at least $1-\delta$ over the random draw of the sample $\mathscr{X}$,*

$$R \leq R_{emp} + 2\beta + (4N\beta + M)\sqrt{\frac{\ln(1/\delta)}{2N}}. \tag{17}$$

This theorem gives tight bounds when the stability β scales as $1/N$[S5]. Applying the regression notion to classification implies neither any misconceptions nor erroneous theory, since classification outcomes are just a looser notion of regression outcomes.

Upper Bounds of Randomized Algorithms

We now examine the stability and error bounds of randomized algorithms. Typical examples include bagging, subbagging and the random forest. This is the follow-up work of Elisseeff in[S7]. We focus on the subbagging variation, whose differences with regard to classical bagging were described earlier in Section S1.1. In a nutshell, a subbagging algorithm uses S subsamples for training, $\mathscr{X}^{(1)}, \ldots, \mathscr{X}^{(S)} \subseteq \mathscr{X}$, of size $p \leq N$ drawn *uniformly and without replacement* (no duplicates allowed). In our algorithm we take $p = \eta N$. The base model that is being subbagged is also referred to as the *base machine*. For instance, in the case of our model, Gentle Boost is used as its base machine, regardless of whether W-CLB or S-CLB is injected.

The upper bounds on the generalization error $R(A, \mathscr{X})$, where A is a symmetric randomized algorithm that uses a training set $\mathscr{X}$, with respect to its outcome $f_{\mathscr{X}}$ and some loss function $\ell(A_{\mathscr{X}}, z)$, for all $z \in \mathscr{Z}$, are the same as the bounds of deterministic algorithms in Theorem S3. This is ensured by Theorem 6[S7]. Therefore, one is interested in only how the stability of a randomized algorithm relates to the stability of its base machine. But, before we proceed, a function f is said to be B-Lipschitzian if for some number B, $|f(x_1) - f(x_2)| \leq B|y_1 - y_2|$.

Supplementary Proposition S1 ((Pointwise) Hypothesis stability of subbagging for regression, Proposition 4.4[S7])**.** *Assume that the loss ℓ is B-Lipschitzian. Let $\Phi_{\mathscr{X}}$ be the outcome of a subbagging algorithm whose base machine is symmetric and has hypothesis (resp. pointwise hypothesis) stability β_p with respect to classification loss ℓ, and subbagging is done by sampling p points without replacement. Then, the random hypothesis (resp. pointwise hypothesis) stability β_N of $\Phi_{\mathscr{X}}$ with respect to the loss function ℓ is bounded by*

$$\beta_N \leq B\beta_p \frac{p}{N}.$$

An example algorithm that exploits this property was proposed in[S10].

The Heaviside and the squared loss, the latter being used by the regression stump, are both 1-Lipschitzian, so we can safely take $B = 1$[S7]. The $\ell_{\gamma=1}$ loss is 2-Lipschitzian according to the original work, Proposition 4.4[S7]. But, ℓ_1 is never used, and we thus have $\beta_N \leq \beta_p p/N$ for the W-CLB flavor. More importantly, the bounds in Theorem S3 and all notions of stability are only dependent on the maximum value M of ℓ and B, but not the nature of ℓ itself.

Supplementary Proposition S2 ((Pointwise) Hypothesis stability of subbagging for classification, Proposition 4.5[S7]). *Let $\Phi_{\mathscr{X}}$ be the outcome of a subbagging algorithm whose base machine is symmetric and has hypothesis (resp. pointwise hypothesis) stability β_p with respect to classification loss, and the subbagging is done by sampling $p < |\mathscr{X}| = N$ points without replacement. Then, for the random hypothesis (resp. pointwise hypothesis) stability β_N of $\Phi_{\mathscr{X}}$ with respect to a 1-Lipschitzian loss function ℓ, the following inequality holds*

$$\beta_N \leq 2\beta_p \frac{p}{N}.$$

Stability of AdaBoost

This part focuses on the interaction of weakness and stability in AdaBoost. To the best of our knowledge, the only existing and established results for the stability of AdaBoost are [S8] and [S11]. The authors in [S8] showed that AdaBoost is almost-everywhere stable. Here, we extend their theory by applying it to Gentle Boost for the purpose of W-CLB and prove that the same stability results prevail. As a corollary, we also infer (and prove) that the stability results of [S8] hold regardless of the risk function minimized by the weak learner in boosting.

Let $\mathscr{H}_N = \{f_q \,|\, q \text{ has support of size at most } N\}$ be the set of all possible classifiers that can be generated by an algorithm A for each possible q, where q is a weight distribution over the training set. $\mathscr{H}$ is the space of weak classifiers, such as the regression or decision stump.

Supplementary Definition S10 (Weakness[S8]). *The weakness of a learning algorithm A, denoted $Weak_D(A)$, is given by*

$$Weak_D(A) = \lim_{N\to\infty} \inf \mathbf{E}_{\mathscr{X}\sim D^N}\left[R_{emp}(\mathscr{H}_N, \mathscr{X})\right].$$

In addition, an algorithm A is said to be weak with respect to a distribution D if $Weak_D(A) > 0$.

This is the same notion as Definition S1. Therefore, any non-perfect predictor is regarded as weak.

Supplementary Theorem S4 (Stability of AdaBoost, Theorem 5.8[S8]). *Suppose that the weak learner A has L_1-stability λ and let $\varepsilon_* = Weak_D(A)/2 > 0$. Then, for sufficiently large N, for all T, AdaBoost in T rounds is (β, δ)-stable (i.e., with probability at least $1-\delta$), where*

$$\beta = \frac{2}{N}\sum_{t=1}^{T} \frac{2^{t^2+1}(\lambda+2)^t}{\varepsilon_*^{2t-1}},$$
$$\delta = e^{-N\varepsilon_*^2/2}. \tag{18}$$

The theorem provided here is adapted to accommodate $y \in \{-1, +1\}$.

S2 Supplementary Discussion

S2.1 Why W-CLB Works

In this section we provide proofs on the effectiveness of W-CLB and describe how it yields a better upper bound on the generalization error. We will very often focus on a single Gentle Boost ensemble trained on some subset $\mathscr{X}^{(j)} \subset \mathscr{X}$ of size $p \leq N$, hence we eliminate the $^{(j)}$ superscript when clear from context to ensure both readability and notation simplicity.

We now consider Gentle Boost's stability. To the best of our knowledge, there have not been established stability notions for Gentle Boost. Theorem 5.8[S8] holds when the class labels are $\{0,1\}$, and each weak learner f_t minimizes the weighted absolute error $\varepsilon_t = w|f_t(\boldsymbol{x}) - y|$. However, the stability of Gentle Boost is slightly different and potentially worse.

Supplementary Theorem S5 (Stability of Gentle Boost)**.** *Suppose that the weak learner A has L_1-stability λ and let $\varepsilon_* = Weak_D(A)/2 > 0$. Then, for sufficiently large N, for all T, Gentle Boost in T rounds is (β,δ)-stable (i.e., with probability at least $1-\delta$), where*

$$\beta = \frac{2}{N}\sum_{t=1}^{T}\frac{2^{t^2+1}(\lambda+4)^t}{\varepsilon_*^{2t-1}},$$
$$\delta = e^{-N\varepsilon_*^2/2}. \tag{19}$$

Our Theorem S5 ultimately states that Gentle Boost is less stable than AdaBoost as a consequence of the squared loss minimized by each constituent weak learner. The following lemma is an immediate consequence.

Supplementary Lemma S3 (Pointwise hypothesis stability of Gentle Boost)**.** *Suppose that for sufficiently large N, for all T, and for some $\mathscr{X} \in \mathscr{Z}^N$, Gentle Boost in T rounds boosts a weak learner with pointwise hypothesis stability β_w. Then, Gentle Boost has pointwise hypothesis stability β with probability at least $1-\delta$ over the random draw of $\mathscr{X}$*

$$\beta = \frac{2}{N}\sum_{t=1}^{T}\frac{2^{t^2+1}\left(\frac{N\beta_w}{2}+4\right)^t}{\varepsilon_*^{2t-1}},$$
$$\delta = e^{-N\varepsilon_*^2/2}. \tag{20}$$

We now have all the necessary tools and knowledge to derive new upper bounds of the generalization error R of Sub-baggged Gentle Boost.

Supplementary Theorem S6 (Generalization error upper bound of Subbagged Gentle Boost)**.** *Assume that the loss function ℓ is B-Lipschitzian, and $0 \leq \ell(\Phi_{\mathscr{X}}, z) \leq M$, for all $z \in \mathscr{Z}$, where $\Phi_{\mathscr{X}}$ is the outcome of a subbagging algorithm whose base machine is Gentle Boost. Next, assume that subbagging is done by sampling S sets of size $p < N$ from some $\mathscr{X} \in \mathscr{Z}^N$ uniformly and without replacement. Now, let the weak learning algorithm A have (pointwise) hypothesis stability β_w with respect to ℓ and let $\varepsilon_* = Weak_D(A)/2 > 0$. Then, for sufficiently large p, for all T, for Subbagged Gentle Boost in T rounds with probability at least $1-\delta$ over the random draw of $\mathscr{X} \sim D^N$,*

$$R(\Phi_{\mathscr{X}}) \leq R_{emp}(\Phi_{\mathscr{X}}) + 2B\beta_p\frac{p}{N} + (4Bp\beta_p + M)\sqrt{\frac{\ln(1/\delta)}{2N}},$$
$$\text{where } \beta_p = \frac{2}{N}\sum_{t=1}^{T}\frac{2^{t^2+1}(\frac{N\beta_w}{2}+4)^t}{\varepsilon_*^{2t-1}},$$
$$\delta = e^{-N\varepsilon_*^2/2}.$$

Theorem S6 can be applied to other kinds of base machines, regardless of their underlying learning algorithm.

Recalling Theorem S6, the upper bound is a sum of two elements: empirical error and overhead cost of stability. The ultimate goal of W-CLB is to reduce both by collaborative exchange of instances that improves pointwise hypothesis stability. We consider the monotonic exponential loss

$$\ell(f,z) = e^{-yf(\boldsymbol{x})}, \quad z \in \mathscr{Z},$$

which is an upper bound of the misclassification loss $\mathbb{1}_{-yf(\boldsymbol{x})\geq 0}$[S9]. Moreover, the exponential loss is minimized by Gentle Boost[S2].

(1) **Lower upper bound.** The term "lower" transliterates to lower empirical error of the complex W-CLB ensemble Φ. The upper bound in Theorem S6 consists of the empirical error plus a numeric term involving stability. In this sense, W-CLB generates an ensemble that has lower empirical error than classical Subbagged Gentle Boost. The effect of lowering the error comes from explicit substitution of small-margin instances. In each of the S Gentle Boost ensembles, there are exactly p_cT regression stumps having a lower empirical (training) error than before. The final output Φ is the outcome of the subbagging ensemble, constructed from S Gentle Boost base machines $F^{(1)},\dots,F^{(S)}$ and is computed by taking the sign of their average. Let $R_{emp}^{W-CLB}(A_{\mathscr{X}})$ denote the empirical error of an algorithm that outputs A on any level of the complex ensemble, trained on $\mathscr{X}$, when W-CLB is injected into the training process, and A is either a regression stump, Gentle Boost or Subbagged Gentle Boost.

Supplementary Theorem S7 (Monotonic minimization of the exponential loss by Gentle Boost)**.** *Let t be the current round of boosting and let $F(\boldsymbol{x})$ be the outcome of a Gentle Boost algorithm from the previous $t-1$ rounds of training on a dataset $\mathscr{X}\in\mathscr{Z}^N$. Assume that $f_t(\boldsymbol{x})$ is the outcome of a real-valued weak learning algorithm, added to the ensemble, then with respect to the exponential loss,*

$$\sum_{i=1}^{p} e^{-y_i(F(\boldsymbol{x}_i)+f_t(\boldsymbol{x}_i))} \leq \sum_{i=1}^{p} e^{-y_iF(\boldsymbol{x}_i)}. \tag{21}$$

In other words, classical boosting procedures yield a lower exponential loss at each round. When W-CLB replaces some $z_i\in\mathscr{X}$ by some z_i' at round t of boosting, we have

$$0\leq y_if_t(\boldsymbol{x}_i)\leq y_i'f_t(\boldsymbol{x}_i'). \tag{22}$$

There are two distinct consequences. First, let the new empirical error of f_t, trained on $\mathscr{X}$, but with respect to $\mathscr{X}'=\mathscr{X}\setminus\{z_i\}\cup\{z_i'\}$ be

$$\varepsilon_{t,partial}' = w_{it}(y_i'f_t(\boldsymbol{x}_i')-1)^2+\sum_{k\neq i}w_{kt}(y_kf_t(\boldsymbol{x}_k)-1)^2. \tag{23}$$

Hence, $\varepsilon_{t,partial}'\leq\varepsilon_t$. In addition, it is clear that the further-training Algorithm 4 cannot result in a worse error. If ε_t' is the empirical error of $f_{t,\mathscr{X}'}$, it follows that

$$\varepsilon_t'\leq\varepsilon_{t,partial}'\leq\varepsilon_t. \tag{24}$$

Second, using Equation (22) also results in a lower empirical error with respect to the total exponential loss, i.e.,

$$e^{-y_i'f_t(\boldsymbol{x}_i')}+\sum_{k\neq i}e^{-y_kf_t(\boldsymbol{x}_k)}\leq\sum_{i=1}^{p}e^{-y_if_t(\boldsymbol{x}_i)}. \tag{25}$$

Equation (25) intuitively leads to a lower Gentle Boost error, and we formally prove that in fact it does.

Supplementary Theorem S8 (W-CLB yields almost-everywhere lower empirical exponential loss of Gentle Boost)**.** *Let t be the current round of Gentle Boost with an outcome $F_{t,\mathscr{X}}(\boldsymbol{x})=\sum_{s=1}^{t}f_{s,\mathscr{X}}(\boldsymbol{x})$ and assume that W-CLB is injected after training $f_{t,\mathscr{X}}(\boldsymbol{x})$, i.e.,between rounds t and $t+1$, yielding $f_{t,\mathscr{X}'}^h$ and $F_{t,\mathscr{X}'}^h$, respectively. Then, with a high probability of ω, W-CLB yields a lower empirical Gentle Boost error $R_{emp}^{W-CLB}(F_{t+1,\mathscr{X}'})$ at round $t+1$ with respect to the exponential loss $\ell(F_{t+1,\mathscr{X}'},z_i), z_i\in\mathscr{X}'$, or*

$$R_{emp}^{W-CLB}(F_{t,\mathscr{X}'}^h+f_{t+1,\mathscr{X}'},\mathscr{X}')\leq R_{emp}(F_{t,\mathscr{X}}+f_{t+1,\mathscr{X}},\mathscr{X}),$$

$$\omega=\mathbf{P}\left[\sum_{\boldsymbol{z}\in\mathscr{X}'}yf_{t+1,\mathscr{X}'}(\boldsymbol{x})\geq\sum_{\boldsymbol{z}\in\mathscr{X}}yf_{t+1,\mathscr{X}}(\boldsymbol{x})\;OR\;\sum_{\boldsymbol{z}\in\mathscr{X}'}yF_{t,\mathscr{X}'}^h(\boldsymbol{x})\geq\sum_{\boldsymbol{z}\in\mathscr{X}}y(F_{t,\mathscr{X}}(\boldsymbol{x})+f_{t+1,\mathscr{X}}(\boldsymbol{x}))\right]. \tag{26}$$

Supplementary Corollary S1. *A single injection of W-CLB at any round $t < T$ of Gentle Boost, where T is the total number of boosting rounds and $\mathscr{X}'$ is the version of the training set at the last round T, it holds with probability ω that*

$$R_{emp}^{W-CLB}(F_T, \mathscr{X}') \leq R_{emp}(F_T, \mathscr{X}),$$

$$\omega = \mathbf{P}\left[\sum_{z \in \mathscr{X}'} y f_{t+1,\mathscr{X}'}(\boldsymbol{x}) \geq \sum_{z \in \mathscr{X}} y f_{t+1,\mathscr{X}}(\boldsymbol{x}) \ \textbf{or} \sum_{z \in \mathscr{X}'} y F^h_{t,\mathscr{X}'}(\boldsymbol{x}) \geq \sum_{z \in \mathscr{X}} y(F_{t,\mathscr{X}}(\boldsymbol{x}) + f_{t+1,\mathscr{X}}(\boldsymbol{x}))\right]. \tag{27}$$

In our experiments, using realistic datasets, we have observed that ω is *very high*. In fact, we have not found a case when either Theorem S8 or Corollary S1 does not hold, hence an "almost-everywhere" improvement by W-CLB. Beside two extremely rare cases when Theorem S8 did not hold promptly at round $t+1$, Corollary S1 was still valid, hence W-CLB might have a delayed effect regarding Theorem S8.

We now consider the empirical error of Subbagged Gentle Boost when S subsamples $\mathscr{X}^{(1)}, \dots, \mathscr{X}^{(S)}$ are uniformly and without replacement drawn from $\mathscr{X}$, the empirical error of $\Phi_{\mathscr{X}}$ cannot be simply averaged over $\mathscr{X}$. The reason is that $\mathscr{X}^{(1)} \cup \mathscr{X}^{(2)} \cup \dots \cup \mathscr{X}^{(S)} \subseteq \mathscr{X}$ and there is a chance that instances from the original $\mathscr{X}$ might not be used for training at all, and are hence "testing" instances with respect to $\Phi_{\mathscr{X}}$. Therefore, the expected empirical error of $\Phi_{\mathscr{X}}$ can be estimated by the first moment of its expected value,

$$\hat{R}_{emp}(\Phi_{\mathscr{X}}) = \hat{\mathbf{E}}_F\left[R_{emp}(\Phi_{\mathscr{X}})\right] = \frac{1}{S}\sum_{j=1}^{S} R_{emp}(F_{\mathscr{X}^{(j)}}), \tag{28}$$

since we do not have the luxury of having all possible Gentle Boost functions over $\mathscr{X}$. Consistently thereafter, we simply use $\hat{R}_{emp}(\Phi_{\mathscr{X}})$ to work with $R_{emp}(\Phi_{\mathscr{X}})$. From Equation (28), it is clear that reducing the average empirical error of individual Gentle Boost ensembles is sufficient to provoke a decrease in the expected empirical error $R_{emp}(\Phi_{\mathscr{X}})$.

(2) **Tighter upper bound.** The second major effect of W-CLB is a tighter upper bound on the generalization error of Subbagged Gentle Boost. W-CLB utilizes pointwise hypothesis stability from Definition S5. From Theorem S6, the stability of the Gentle Boost base machine in subbagging was expressed through the stability of individual weak learners – the T regression stumps. It delivers a view at the lowest level of the complex ensemble in terms of stability. Consequently, whenever a single regression stump manifests better stability, the effect is transmitted up to the highest level at $\Phi_{\mathscr{X}}$, which combines ST more stable regression stumps.

From the W-CLB steps described in Algorithm 3, for the j-th Gentle Boost ensemble at round t, $j \in [1, S]$, performs a two-step replacement of some $z_i \in \mathscr{X}^{(j)}$ by $z'_i \in \mathscr{X}^{(k)}, k \neq j$, such that $\mathscr{X}^{(j)\prime} = \mathscr{X}^{(j)} \setminus \{z_i\} \cup \{z'_i\}$:

i) Find $z_i = \arg\min_i y_i f_{t,\mathscr{X}^{(j)}}(\boldsymbol{x}_i)$

ii) Replace z_i by z'_i, where $y'_i f_{t,\mathscr{X}^{(j)}}(\boldsymbol{x}'_i) \geq y_i f_t(\boldsymbol{x}_i)$.

Ultimately, the new z'_i has a larger margin with respect to $f_{t,\mathscr{X}^{(j)}}$.

Here, we will use the exponential loss again, but this time with respect to the weak learner f in the j-th Gentle Boost ensemble, $j \in [1, S]$ (the boosting round is clear from context for now). Therefore,

$$\ell(f_{\mathscr{X}^{(j)}}, z) = e^{-y f_{\mathscr{X}^{(j)}}(\boldsymbol{x})}, \quad z \in \mathscr{Z}. \tag{29}$$

We are primarily interested in the expected absolute loss difference from Equation (8), and the following proposition claims why increasing the margins in the training set potentially improves stability in the general case.

Supplementary Proposition S3. *Let f be the outcome of a real-valued classification algorithm, trained on a dataset $\mathscr{X}$ and let ℓ be the exponential loss. Then for any two correctly classified training instances $z_i, z_k \in \mathscr{X}$, such that $0 \leq y_i f_{\mathscr{X}}(\boldsymbol{x}_i) \leq y_k f_{\mathscr{X}}(\boldsymbol{x}_k)$,*

$$|\ell(f_{\mathscr{X}}, z_i) - \ell(f_{\mathscr{X}^{\setminus i \cup z}}, z_i)| \geq |\ell(f_{\mathscr{X}}, z_k) - \ell(f_{\mathscr{X}^{\setminus k \cup z}}, z_k)|, \quad z \in \mathscr{Z}. \tag{30}$$

Proposition S3 implies a lower expected value of the absolute loss difference in Definition S5. In essence, it decreases the lower bound of pointwise hypothesis stability β_w of the weak learner. Although better stability for the replaced instance is guaranteed, the absolute loss differences at the other positions potentially perturb, and for this reason we stabilize that by the Further-Training algorithm.

A logical arousing question related to the counterintuitivity of W-CLB is "Why not replace negative margins by positive ones?" The answer consists of two parts:

Conservativism. The idea comes from the Gentle Boost algorithm itself. For instance, Gentle Boost (Algorithm 1) is a conservative version of Logit Boost, which displays big minimization steps at pure regions, or more precisely, learns the training data in a quicker fashion[S2]. We apply the same conservative idea here; replacing a negative margin by a positive one has an overwhelming effect on reducing the empirical error, but potentially imposes overfitting. Mild improvements as those of W-CLB are clearly both effective and sufficiently strong to improve the generalization error.

Challenge. All incorrectly classified instances remain in the subset. With this, we force the Gentle Boost ensemble to accept the challenge of learning them, instead of providing it with instances it easily classifies correctly. In fact, W-CLB makes a trade-off between improving knowledge and forcing, while prevents faking knowledge. This claim is also numerically confirmed. The further-training Algorithm 4 operating immediately after W-CLB ensures that the regression stump at which W-CLB has been injected either preserves its previous optimal parameters, or gets better ones. Therefore, it ensures a greater or equal sum of the instance margins in the modified training subset at round t. An immediate consequence is the weights-normalization constant $Z_t^{W-CLB} < Z_t$. If $f^h_{t,\mathscr{X}'} = f_{t,\mathscr{X}'}$, then each incorrectly classified instance is heavier than its counterpart in round $t+1$ when W-CLB is not injected. On the other hand, each new instance added by W-CLB gets a lower weight than its counterpart at round $t+1$. Thus, it makes sense to ensure that all incorrectly classified instances get larger weights in the next iteration. Moreover, the total increase of the weights of misclassified instances is equal to the total decrease of the weights of newly added instances with larger margins. Increasing negative margins with W-CLB does not guarantee this, i.e.,makes the next regression stump "forget" about some of the incorrect predictions by reducing their weights, which is indeed considered as faking knowledge. These claims are completely true when $f^h_{t,\mathscr{X}'} = f_{t,\mathscr{X}'}$, and are conversely slightly looser.

We now analyze the worst-case performance of Subbagged Gentle Boost with W-CLB and compare it to Gentle Boost. The Regression Stump algorithm, described in Algorithm 2, has $O(d^2N^2)$ worst-case performance, where N is the size of the training set and d is the number of features, or the dimensionality of the population space. Henceforth, Gentle Boost in T rounds of boosting has $O(d^2N^2T)$ worst-case performance, based on Algorithm 1.

Analogously, the Subbagged Gentle Boost algorithm has $O(d^2\eta^2N^2TS)$ worst-case complexity. It is worth noting that this performance is usually better than Gentle Boost's performance because it involves portions of the training data. Injecting W-CLB here, leads to the following worst-case performance of our algorithm:

$$O\left(d^2\eta^2N^2TS + p_cT(S^2\eta Nn_{exc} + d^2n_{exc}^2)\right),$$

since it takes $O(S^2\eta Nn_{exc})$ to exchange instances using Algorithm 3 and apply Algorithm 4 in $O(d^2n_{exc}^2)$ as a final step.

In our experimental evaluation of the proposed method we have observed that the computational overhead imposed by W-CLB often substantiates the possibility of better performance than T rounds of gentle boosting over the whole training set $\mathscr{X}$.

S2.2 Why S-CLB Works

In order to explain how stability theory can be applied for the purpose of improving the generalization performance of our model, let us assume that the data in $\mathscr{X}$ is used for training a model based on subbagging of Gentle Boost ensembles, i.e.,a Subbagged Gentle Boost. Moreover, let us assume that our proposed random sampling strategy presented in Section S1.1 is used to divide $\mathscr{X}$ into S different data subsets $\mathscr{X}^{(1)}, \mathscr{X}^{(2)}, \ldots, \mathscr{X}^{(S)}$ of equal size and multiple Gentle Boost ensembles are trained such that $\mathscr{X}^{(j)}$ plays the role of a training set for the j-th ensemble, for all $j = 1, \ldots, S$. Since a Gentle Boost ensemble is also a classifier itself, produced by an ensemble method, essentially its training can be seen as usage of a learning algorithm A which outputs a hypothesis based on the knowledge gathered from the training data. Analogously, in our case, the training of each Gentle Boost ensemble is conducted by the usage of the Gentle Boost algorithm A which returns the hypothesis function $F_{\mathscr{X}^{(j)}}$. As in Section S1.3.1, we adopt $F_{\mathscr{X}^{(j)}} \equiv F^{(j)}$. It is important that in order to apply the stability theory to provide bounds on the generalization error of a given classification model, the adequate learning algorithm must be symmetric with respect to the data in its supplied training set. It is known that boosting algorithms are symmetric, which approves the appliance of stability theory in our case. So, if a Gentle Boost ensemble is trained on each subset $\mathscr{X}^{(j)}$ by means of A whose outcome $F^{(j)}$ obviously does not depend on the instance order in $\mathscr{X}^{(j)}$, then Theorem S3, which was initially introduced by Bousquet and Elisseeff as Theorem 12[S5], can be applied to bound the generalization error of A. But, as stated in the theorem, the suggested bounds hold only if the algorithm used for training each base machine within the subbagging scheme is a real-valued one.

This means that $F^{(j)}$ must be a real-valued function such that the label of a given instance $\boldsymbol{x}$ is predicted by taking the sign of $F^{(j)}(\boldsymbol{x})$, for each $j = 1, \dots, S$, which was defined in Equation (**??**). Note that according to this definition, $F^{(j)}(\boldsymbol{x})$ does not directly represent the label predicted on $\boldsymbol{x}$ by the j-th ensemble, but what it represents is the confidence that the j-th ensemble has in this prediction. This way of defining $F^{(j)}(\boldsymbol{x})$ enables the usage of the classification loss. As to the decision-making, the final label predicted by the j-th ensemble is sign$[F^{(j)}(\boldsymbol{x})]$.

As stated in Section 4.2.2[S5], a good real-valued classification algorithm is one that produces outputs whose absolute values truly represent the confidence they have in a certain prediction. Considering this and the nature of the boosting algorithms, we can see that the real-valued output $F^{(j)}(\boldsymbol{x})$ is intentionally chosen such that, for any instance $\boldsymbol{x}$, $|F^{(j)}(\boldsymbol{x})|$ is a true representative of the confidence for predicting the instance label sign$[F^{(j)}(\boldsymbol{x})]$. Moreover, choosing $F^{(j)}(\boldsymbol{x})$ as in Equation (**??**) makes the j-th Gentle Boost ensemble eligible for performance evaluation in terms of classification loss. Consequently, both classification and uniform stability can be used to measure the stability of each ensemble. The theorem that follows provides an upper bound on the generalization error of our model regardless of the stability measure choice.

Supplementary Theorem S9 (Classification-loss-oriented upper generalization error bound of Subbagged Gentle Boost)**.** *Let $\ell_T(\Phi_{\mathscr{X}}, z)$ be a T-Lipschitzian classification loss function, for all $z \in \mathscr{Z}$, where $\Phi_{\mathscr{X}} : \mathbb{R}^d \to \mathbb{R}$ is the outcome of a real-valued Subbagged Gentle Boost model consisted of S base Gentle Boost ensembles, while each one of them is trained using $T > 1$ weak learners. Then, for any $N \geq 1$, and any $\delta \in (0,1)$, with probability at least $1-\delta$ over the random draw of a training set $\mathscr{X}$,*

$$R(\Phi_{\mathscr{X}}, \mathscr{X}) \leq R^T_{emp}(\Phi_{\mathscr{X}}, \mathscr{X}) + 4\eta\beta_p + (8\eta N\beta_p + 1)\sqrt{\frac{ln(1/\delta)}{2N}}, \tag{31}$$

where β_p is the stability of the base Gentle Boost ensemble with respect to ℓ_T, and $\eta = |\mathscr{X}^{(j)}|/|\mathscr{X}|$.

The following discussion provides an explanation about the reasons for proposing the S-CLB procedure; in other words, it simply explains why S-CLB works and how this approach contributes to lowering and potentially tightening the upper bound of the generalization error of the whole model. This is done by separately analyzing the rationality of each step within the procedure, thus resulting in a single fused discussion.

Assuming that the j-th and the k-th ensemble are about to collaborate at the τ-th iteration, first they must satisfy the collaboration criterion defined in Step I. For this purpose, the margins of all instances in $\mathscr{X}^{(j\backslash k,\tau)}$ and $\mathscr{X}^{(k\backslash j,\tau)}$ are sorted separately. It is known that, given an instance-label pair $z = (\boldsymbol{x}, y)$, the generalization error R of a boosting ensemble trained on a sample whose examples are chosen independently at random according to a distribution D, is defined as the probability of $\boldsymbol{x}$ to have a negative margin, i.e.

$$\mathbf{P}_D\left[yF(\boldsymbol{x}) \leq 0\right] = R.$$

Since the j-th and the k-th ensemble are trained using a boosting algorithm (in this case, Gentle Boost), i.e.,they are essentially boosting ensembles, by increasing the margins of the instances in the relative complements $\mathscr{X}^{(j\backslash k,\tau)}$ and $\mathscr{X}^{(k\backslash j,\tau)}$, theoretically, the generalization errors of these ensembles should be reduced. This is the case because $\mathscr{X}^{(j\backslash k,\tau)} \subseteq \mathscr{X}^{(j,\tau)}$ and $\mathscr{X}^{(k\backslash j,\tau)} \subseteq \mathscr{X}^{(k,\tau)}$, so by increasing the margins of the instances in the relative complements, the margins of some instances from the training sets are increased as well. But, these datasets may also contain instances whose margins already have sufficiently large values and any increase of these values is considered to be of low importance and priority. This means that maximizing an already large margin would cause a far less significant change in an ensemble's generalization performance than maximizing the minimal margins. However, boosting algorithms mostly produce ensembles with large minimum margins[S12], so even after all margins gain relatively large values, further increasing them is still considered to be a positive change towards reducing the ensemble's generalization error as Breiman states in[S4]. Therefore, the concept of margins is used to measure the contribution of a given instance to the reduction of the generalization error of its parent ensemble, thus reducing the generalization error of the whole model. In our case, this is achieved by choosing the top $n^{(\tau)}_{exc}$ instances from the sorted margin sequences with respect to $F^{(\tau)}_{\mathscr{X}^{(j,\tau)}}$ and $F^{(\tau)}_{\mathscr{X}^{(k,\tau)}}$, separately, as the ones which are going to be exchanged in Step II.

After selecting the instances with the minimal margins from both $\mathscr{X}^{(j\backslash k,\tau)}$ and $\mathscr{X}^{(k\backslash j,\tau)}$, some or all of the top $n^{(\tau)}_{exc}$ instances with minimal margins from the former are swapped with the corresponding ones from the latter. Swapping of the instances with minimal margins is chosen to be the method for exchanging training information between the j-th and the k-th Gentle Boost ensemble for two reasons:

- The first one is the need of keeping consistency with regard to the concepts from the stability theory used to derive the model's generalization error bound in Theorem S9. More precisely, the bound holds only if $\eta = |\mathscr{X}^{(s,\tau)}|/|\mathscr{X}|$ for

each $s = 1, \ldots, S$ and $\tau = 1, \ldots, \mathscr{T}$. This means that each instance which is going to be removed from $\mathscr{X}^{(j\setminus k,\tau)}$ must be replaced by only one instance from $\mathscr{X}^{(k\setminus j,\tau)}$ in order to sustain the original cardinality of $\mathscr{X}^{(j,\tau)}$ and $\mathscr{X}^{(k,\tau)}$, as well as to keep the equal subset size constraint satisfied.

- The second reason is supported by the fact that only instances from the relative complements $\mathscr{X}^{(j\setminus k,\tau)}$ and $\mathscr{X}^{(k\setminus j,\tau)}$ having the minimal margins are eligible for swapping. Considering this, it is clear that each instance from $\mathscr{X}^{(j,\tau)}$ is going to be replaced by one which is contained in $\mathscr{X}^{(k,\tau)}$, but not in $\mathscr{X}^{(j,\tau)}$, and the other way around. This swapping principle guarantees that no instance will be allowed to be duplicated within a single training subset.

As to the instance swapping itself, it is conducted using the set of all potential swapping orders $\mathscr{S}^{(j,k,\tau)}$ of at most $n_{exc}^{(\tau)}$ swapping pairs. But the optimal swapping order may not be the one according to which $n_{exc}^{(\tau)}$ instances from $\mathscr{X}^{(j\setminus k,\tau)}$ are swapped with exactly $n_{exc}^{(\tau)}$ instances from $\mathscr{X}^{(k\setminus j,\tau)}$, but rather it could be just one that includes swapping of less than $n_{exc}^{(\tau)}$ instances from both sets. Hence, $\mathscr{S}^{(j,k,\tau)}$ contains all swapping orders of n swapping pairs, for each $n = 1, \ldots, n_{exc}^{(\tau)}$. Moreover, an additional driver for generating $\mathscr{S}^{(j,k,\tau)}$ is the combinatorial nature of Step II, as well as the fact that without using $\mathscr{S}^{(j,k,\tau)}$ the training process would gain vast computational complexity. A worst-case scenario would be one in which $\mathscr{X}^{(j,\tau)}$ and $\mathscr{X}^{(k,\tau)}$ are disjoint, i.e.,$\mathscr{X}^{(j,\tau)} \cap \mathscr{X}^{(k,\tau)} = \varnothing$, while the maximal number of instances allowed to be exchanged equals the number of instances allocated for each data subset. This results in a calculation of all possible swapping orders of at most ηN instances and searching for the optimal one by exchanging instances according to each one of these orders. Considering the number of possible swapping orders in this scenario $n_{swap}^{(\tau)} = \sum_{n=1}^{\eta N} \binom{\eta N}{n}^2$, for a large value of ηN, i.e.,in the case of massive data subsets, swapping instances between ensembles according to these orders and retraining them afterwards would be simply infeasible. On the other hand, just a few instances swapped between the ensembles can already make an improvement of their individual ability to generalize. So, this way the improvement of the overall generalization performance is not achieved by increasing the number of swapping orders for examination per iteration, but rather by employing a larger number of collaborations (iterations) between the base ensembles. However, a compromise regarding the generation of $\mathscr{S}^{(j,k,\tau)}$ can still be made in terms of the training algorithm's execution time. Since $n_{exc}^{(\tau)} = \min\,\{n_{exc}, |\mathscr{X}^{(j\setminus k,\tau)}|, |\mathscr{X}^{(k\setminus j,\tau)}|\}$, meaning that $n_{exc}^{(\tau)} \leq n_{exc}$, $\left\{\mathscr{S}_{n,p_j,p_k}^{(j,k,\tau)}\right\}_{p_j=1}^{\binom{n_{exc}^{(\tau)}}{n}}{}_{p_k=1}^{\binom{n_{exc}^{(\tau)}}{n}}$ remains unmodified for each $n = 1, \ldots, n_{exc}^{(\tau)}$ and throughout all $\tau = 1, \ldots, \mathscr{T}$. Therefore, these sets can be generated in advance and later used as "lookup tables" during the whole procedure. By performing this simple and yet useful technical trick, a decent complexity reduction can be achieved.

At last, Step III is the one which determines whether a swapping order is the optimal one or not. This is done by measuring the distance defined in Section S1.3.2 (Step III) and comparing its value with the one obtained before instances were exchanged according to a certain swapping order. As to the distance measure itself, it is defined in a conservative fashion such that an information exchange is considered to be successful only if its value is not worsening after the exchange has been made. The contribution of this collaboration-regulatory principle is shown through several mathematical statements that follow.

Supplementary Theorem S10 (Monotonicity of the empirical error estimate)**.** *Let $\Phi_{\mathscr{X}} : \mathbb{R}^d \to \mathbb{R}$ be the outcome of a real-valued collaborative Subbagged Gentle Boost model trained on $\mathscr{X}$. If S-CLB is used as a method for collaboration between its constituent Gentle Boost ensembles, then $R_{emp}^{T}(\Phi_{\mathscr{X}}^{(\tau)}, \mathscr{X})$, as a function of τ, monotonically decreases as the value of τ increments by one.*

Note that the proof of the above theorem states that $R_{emp}^{T}(\Phi_{\mathscr{X}}^{(\tau)}, \mathscr{X})$ is a monotonically decreasing function of τ, but the step by which its value decreases between iteration τ and $\tau + 1$ is determined by the maximal error distance measured at iteration τ, for each $\tau = 1, \ldots, \mathscr{T} - 1$.

Supplementary Corollary S2 (Almost-everywhere lower classification-loss-oriented upper bound of Subbagged Gentle Boost)**.** *The cumulative S-CLB approach yields an approximately lower upper bound on the generalization error of $\Phi_{\mathscr{X}}$ with high probability of*

$$\omega = \sum_{j=2}^{S} \sum_{k=1}^{j-1} \mathbf{P}\left[I_{p^*}^{(j,k,(j(j-1))/2-j+k)}\right],$$

if its base machine is already stable, i.e.

$$
\begin{aligned}
R(\Phi_{\mathscr{X}}, \mathscr{X}) &\overset{Trm.\ S9}{\leq} R^{T}_{emp}(\Phi_{\mathscr{X}}, \mathscr{X}) + 4\eta\beta_p + (8\eta N\beta_p + 1)\sqrt{\frac{ln(1/\delta)}{2N}} \\
&= R^{T}_{emp}(\Phi^{(0)}_{\mathscr{X}}, \mathscr{X}) + 4\eta\beta_p + (8\eta N\beta_p + 1)\sqrt{\frac{ln(1/\delta)}{2N}} \\
&\gtrapprox R^{T}_{emp}(\Phi^{(\mathscr{T})}_{\mathscr{X}}, \mathscr{X}) + 4\eta\beta_p + (8\eta N\beta_p + 1)\sqrt{\frac{ln(1/\delta)}{2N}} \\
&= R^{T}_{emp}(\Phi^{S-CLB}_{\mathscr{X}}, \mathscr{X}) + 4\eta\beta_p + (8\eta N\beta_p + 1)\sqrt{\frac{ln(1/\delta)}{2N}}.
\end{aligned}
$$

Inequality holds if β_p has a constant value or in case when the empirical error decrease is more significant than the potential increase in the value of the stability measure.

Supplementary Proposition S4. *Let $F_{T,\mathscr{X}}$ be the outcome of a Gentle Boost algorithm trained on $\mathscr{X}$ in T boosting rounds that acts like a base machine of a real-valued Subbagged Gentle Boost. Then, given two positive integers T' and T'' such that $T' \leq T''$, for any instance $z_i = (\boldsymbol{x}_i, y_i) \in \mathscr{X}$ that is correctly classified by both $F_{T',\mathscr{X}}$ and $F_{T'',\mathscr{X}}$,*

$$
|\ell_{T'}(F_{T',\mathscr{X}}, z_i) - \ell_{T'}(F_{T',\mathscr{X}^{\setminus i \cup z}}, z_i)| \geq |\ell_{T''}(F_{T'',\mathscr{X}}, z_i) - \ell_{T''}(F_{T'',\mathscr{X}^{\setminus i \cup z}}, z_i)|, \quad z \in \mathscr{Z}.
$$

Similarly to Proposition S3, Proposition S4 also entails a lower expected absolute loss difference, but unlike the former which refers to the lowest level of the Subbagged Gentle Boost, the latter may contribute to decreasing the lower bound of the pointwise hypothesis stability β_p of the Gentle Boost base machine. The above proposition also suggests that, for a large value of $\mathscr{T}$, even after $R^{T}_{emp}(\Phi^{(\mathscr{T})}_{\mathscr{X}}, \mathscr{X})$ reaches 0, the base machine's pointwise hypothesis stability may continue to improve. Moreover, due to the boosting nature of the underlying base machines, for a sufficiently large number of boosting iterations T per each, the decrease of $R^{T}_{emp}(\Phi^{(\mathscr{T})}_{\mathscr{X}}, \mathscr{X})$ will become more significant that the change in the stability measure's value. In other words, the stronger the Gentle Boost machine is, the stabler it gets.

A brief note on the complexity of S-CLB. The worst-case performance of S-CLB can be easily derived in a bottom-up fashion. As stated in W-CLB's complexity analysis part at the end of Section S2.1, a regression stump (Algorithm 2) is trained in $O(d^2N^2)$, meaning that T rounds of gentle boosting a regression stump take $O(d^2N^2T)$. Now, subbagging S Gentle Boost ensembles leads to a worst-case complexity of

$$
O\left(d^2\eta^2N^2TS\right), \tag{32}
$$

while the collaboration between each pair of ensembles has

$$
O\left(\sum_{n=1}^{n^{(\tau)}_{exc}} \binom{n^{(\tau)}_{exc}}{n}^2 + 2d^2\eta^2N^2T\right). \tag{33}
$$

The first term in the collaboration complexity refers to the instance exchange process between a pair of ensembles, while the second one represents the complexity of retraining both ensembles after instances are being exchanged. By combining (32) and (33), while considering that S-CLB is conducted through $\mathscr{T} = (S-1)S/2$ consecutive iterations, for the worst-case complexity of a S-CLB-guided collaborative Subbagged Gentle Boost we get the following

$$
O\left(d^2\eta^2N^2TS + \sum_{\tau=0}^{((S-1)S/2)-1}\left(\sum_{n=1}^{n^{(\tau)}_{exc}} \binom{n^{(\tau)}_{exc}}{n}^2 + 2d^2\eta^2N^2T\right)\right). \tag{34}
$$

S3 Supplementary Data Description

All nine datasets used throughout the experimental stage of this research encompass real-world tasks. The description of each one of them is provided below.

- The Australian Credit Approval dataset contains data about credit card applications. It was initially provided by a large bank whose name is confidential. Each instance in the dataset represents an application for a credit card consisted of customer information. The challenge is to classify a customer as (in)eligible for a credit card approval. It is worth mentioning that this dataset is also considered as a noisy one.

- The Breast Cancer Wisconsin dataset concerns cancer diagnosis. More precisely, it contains data regarding patients having a breast tumour that is needed to be used in order to predict whether a patient's tumour is non-cancerous or cancerous, i.e.,benign or malignant, respectively. The data was collected in portions, periodically, by Dr. William H. Wolberg at the University of Wisconsin Hospitals which were later aggregated in a single dataset.

- The Pima people (American Indians originating from southern Arizona) were examined for the presence of diabetes and their patient records were assembled in the Diabetes dataset. All patients were females and all of them were at least 21 years old. The diagnostic, binary-valued variable representing the presence of diabetes is investigated to forecast the onset of diabetes mellitus in this high risk population of Pima Indians.

- The Statlog (Heart) is a scanty dataset containing medical data which can be used to determine an absence or presence of a heart disease.

- The Ionosphere dataset is collected by a system in Goose Bay, Labrador, targeting free electrons in the Earth's ionosphere. It consists of radar data used to classify radar returns from the ionosphere as either "Good" or "Bad". Good radar returns are those that return evidence of some type of structure in the ionosphere, while ones that do not are considered bad.

- The BUPA Medical Research Ltd. used blood tests' records to construct the Liver Disorders dataset, such that each data instance refers to a record of a single male individual. These blood tests were sensitive to liver disorders caused by an excessive alcohol consumption.

- The Lung Cancer data concerns classification between malignant pleural mesothe-lioma (MPM) and adenocarcinoma (ADCA) of the lung. There are 181 tissue samples (31 MPM and 150 ADCA), such that each sample is described by 12533 genes.

- The Mammographic Mass dataset incorporates data generated from mammogram screening for breast cancer diagnosis. A BI-RADS (Breast Imaging Reporting and Data System) assessment, the patient's age and three BI-RADS attributes define a single data instance. Each instance is also associated with the ground truth (the severity field). The primary goal is to use this information to predict the severity of a patient's mammographic mass lesion, i.e.,to determine whether it is a benign or a malignant one. Moreover, these predictions can be also used to calculate the sensitivities and associated specificities which indicate how well a CAD system performs compared to the radiologists.

- The Congressional Voting Records dataset, as its title implies, contains votes for U.S. House of Representatives Congressmen from the second session of the 98th Congress in 1984. Essentially, the problem comes down to classifying each voting record as "republican" or "democrat" based on the 16 additional votes identified by the Congressional Quarterly Almanac.

S4 Supplementary Tables

Supplementary Table S1. Summary of the nine datasets.

Dataset	# Instances	# Attributes
Australian	690	14
Breast Cancer	699	10
Diabetes	768	8
Heart	270	13
Ionosphere	351	34
Liver Disorders	345	7
Lung Cancer	181	12533
Mammographic	961	6
Vote	435	16

Supplementary Table S2. Table of input parameters.

Dataset	Subbagging		Gentle Boost	SBGB + WCLB				SBGB + SCLB		
	S	η	T	S	η	p_c	n_{exc}	T	η	n_{exc}
Australian	7	0.1	34	3	0.4	0.5	1	25	0.3	2
Breast Cancer	11	0.5	28	5	0.2	0.05	5	35	0.6	1
Diabetes	32	0.1	21	15	0.5	0.2	5	15	0.2	3
Heart	6	0.2	18	3	0.4	0.5	1	3	0.1	1
Ionosphere	2	0.7	46	20	0.3	0.1	5	15	0.9	3
Liver Disorders	38	0.4	10	40	0.1	0.05	1	10	0.8	1
Lung Cancer	14	0.9	1	15	0.9	1	2	5	0.95	1
Mammographic	33	0.1	15	15	0.6	0.04	30	40	0.5	1
Vote	1	0.1	4	10	0.4	0.25	5	5	0.4	2

S4.1 Parameter Value Selection

The parameter values shown in Table S2 were chosen using an intuitive trial-and-error approach that was for most part driven by the dataset characteristics. We use three major starting points to choose these values; the first baseline to choose η is the dataset size. We assess this value by choosing larger values for small training sets, and vice versa. For instance, Mammographic is the largest one, implying smaller values for η, while for Lung Cancer, being the smallest dataset by size, we use significantly larger values approaching 1. Next, we assess the parameter values by the number of tentative collaborations that were observed as successful, again using a trial-and-error approach, chosen along with n_{exc}. Lastly, η was chosen to improve the robustness in terms of T and S for W-CLB and S-CLB, respectively. In the case of W-CLB, a larger value of T increases the number of collaborations, while for S-CLB this is done by S. In other words, T and S define the collaboration timeframe. Sections S2.1 and S2.2 provide a complexity analysis in detail. For the effect that the model parameters have on its complexity, an additional analysis across different parameter sets was performed to examine how the computational operation count changes with different values of the most significant parameters. The analysis is recapitulated in Figures S1 and S2 for W-CLB and S-CLB, respectively. We anticipate applying parameter meta-optimization and search strategies (e.g., grid search) in our future work.

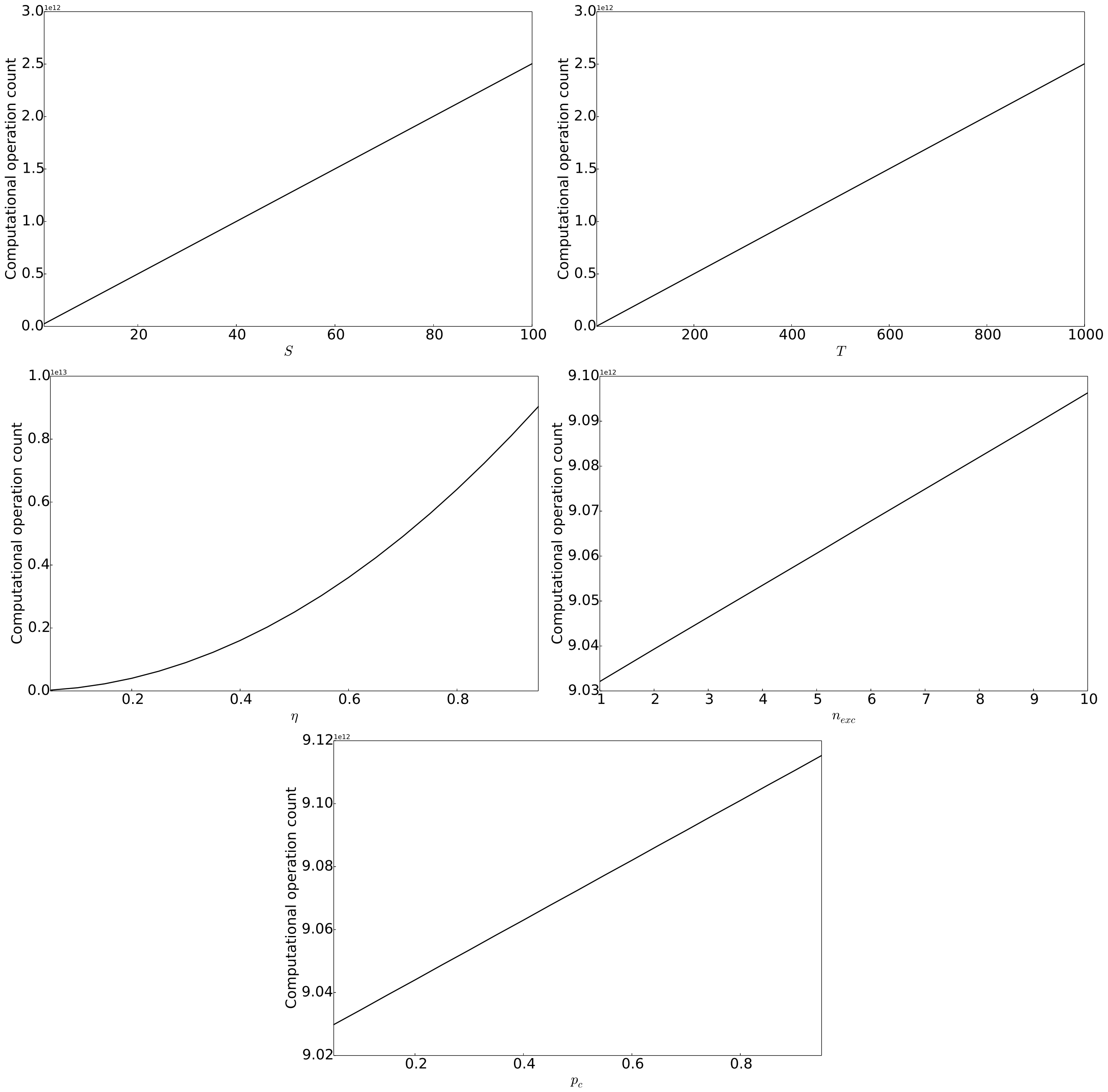

Supplementary Figure S1. Computational operation count of a W-CLB-driven collaborative Subbagged Gentle Boost, produced by different parameter values.

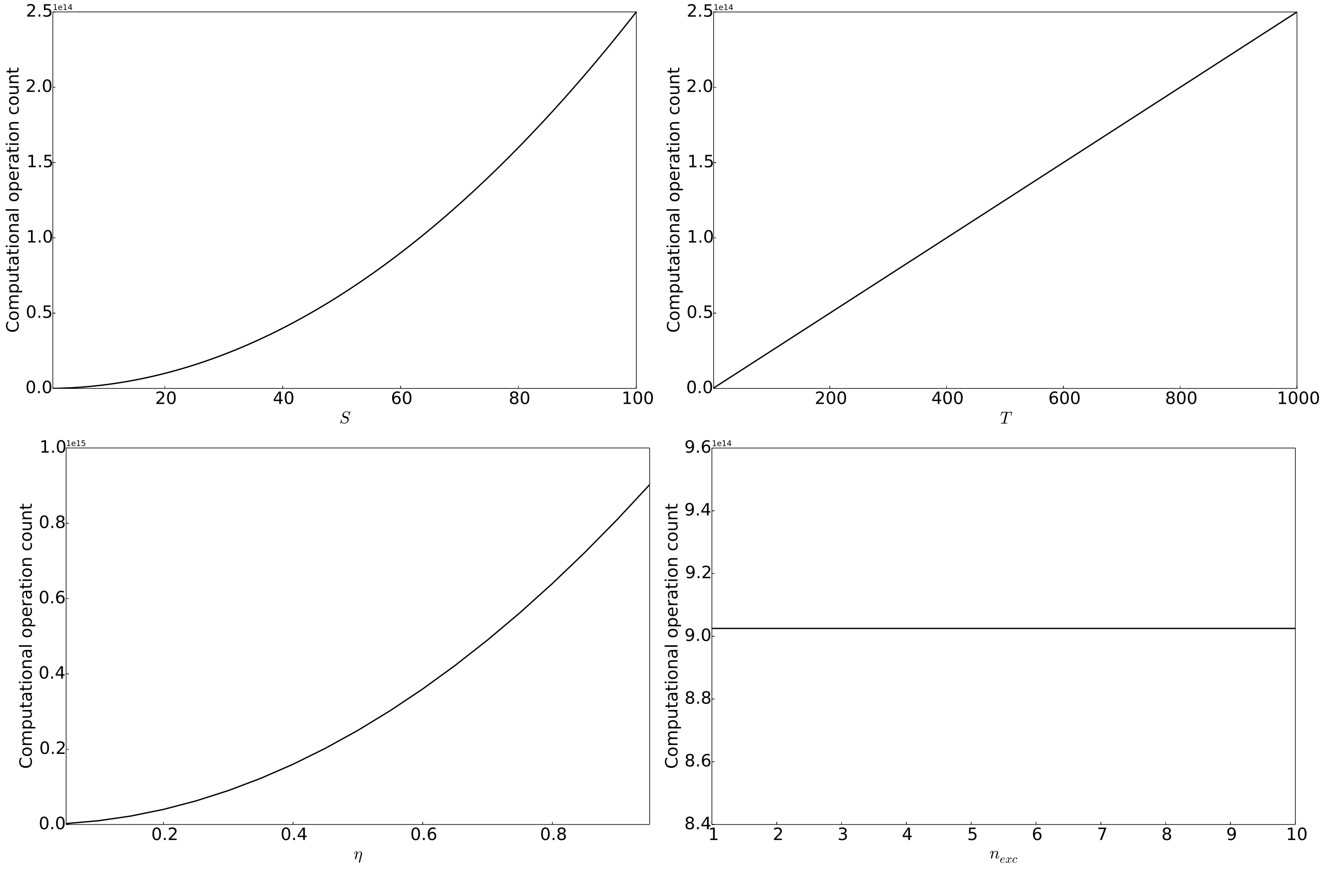

Supplementary Figure S2. Computational operation count of a S-CLB-driven collaborative Subbagged Gentle Boost, produced by different parameter values.

S5 Supplementary Proofs

Proof of Supplementary Theorem S1

Proof. Start by reinterpreting the theorem: The system of equations obtained by setting the partial derivatives of ε_t w.r.t a and b to zero has a unique solution. Let $\boldsymbol{A}$ be the coefficient matrix of the system

$$\frac{\partial \varepsilon_t}{\partial a} = 0 \wedge \frac{\partial \varepsilon_t}{\partial b} = 0.$$

By calculating the partial derivatives and rearranging the terms after some algebraic operations, we obtain the coefficient matrix

$$\boldsymbol{A} = \begin{pmatrix} \langle \boldsymbol{w}, \mathbb{1}_{k,\tau} \rangle & \langle \boldsymbol{w}, \mathbb{1}_{k,\tau} \rangle \\ \langle \boldsymbol{w}, \mathbb{1}_{k,\tau} \rangle & \|\boldsymbol{w}\|_1 \end{pmatrix}.$$

Now, applying Cramer's Rule yields that the system has exactly one unique solution if and only if $\det(\boldsymbol{A}) \neq 0$. We now have to show that $\det(\boldsymbol{A}) \neq 0$.

$$\begin{aligned} \det(\boldsymbol{A}) &= \begin{vmatrix} \langle \boldsymbol{w}, \mathbb{1}_{k,\tau} \rangle & \langle \boldsymbol{w}, \mathbb{1}_{k,\tau} \rangle \\ \langle \boldsymbol{w}, \mathbb{1}_{k,\tau} \rangle & \|\boldsymbol{w}\|_1 \end{vmatrix} \\ &= \langle \boldsymbol{w}, \mathbb{1}_{k,\tau} \rangle \left(\|\boldsymbol{w}\|_1 - \langle \boldsymbol{w}, \mathbb{1}_{k,\tau} \rangle \right) \\ &= \langle \boldsymbol{w}, \mathbb{1}_{k,\tau} \rangle \left(1 - \langle \boldsymbol{w}, \mathbb{1}_{k,\tau} \rangle \right), \end{aligned}$$

since the instance weights are normalized such that $\|\boldsymbol{w}\|_1 = \sum_{i=1}^N |w_i| = \sum_{i=1}^N w_i = 1$. For any $\tau \in \mathscr{T}$, it follows that

1. $\langle \boldsymbol{w}, \mathbb{1}_{k,\tau} \rangle \neq 0$ for any $k \in [1,d]$ because at least one indicator must be equal to 1, which holds even when τ is the largest (last) element of any ℓ_k in the set $\mathscr{T}$. Additionally, the instance weights are always positive and nonzero.
2. $\langle \boldsymbol{w}, \mathbb{1}_{k,\tau} \rangle \neq 1$ for any $k \in [1,d]$ because at least one indicator must be equal to 0, which holds even when τ is the least (first) element of any ℓ_k in the set $\mathscr{T}$.
3. Consequently, $0 < \langle \boldsymbol{w}, \mathbb{1}_{k,\tau} \rangle < 1$.

It follows from the definition of the set $\mathscr{T}$ that $\det(\boldsymbol{A}) \neq 0$. □

Proof of Supplementary Theorem S5

Proof. Let $\mathbf{P} = [p_1\, p_2 \ldots p_N]^T$ and $\mathbf{P}' = [p'_1\, p'_2 \ldots p'_N]^T$ be two weight distributions of a training set $\mathscr{X}$ of size N. Finally, let $y \in \{-1,+1\}$.

Now, let a be an unweighted cost $c(f,y)$ for the loss $\ell(f,z)$ of f on y with respect to p, at an arbitrary round of boosting, and $z = (\boldsymbol{x}, y) \in \mathscr{Z}$. Then,

$$a_i = (f_{\mathscr{X}}(\boldsymbol{x}_i) - y_i)^2,$$
$$\varepsilon = \sum_i p_i a_i.$$

We now use Inequality (6) in Lemma 5.3[S8] and reproduce it to accommodate Gentle Boost. From the L_1-stability λ of the weak learner, we have

$$|a_i - a'_i| = |\ell(f_{\mathscr{X}}, z_i) - \ell(f'_{\mathscr{X}}, z_i)| \leq \lambda \|p - p'\|.$$

Thus, it follows that

$$\begin{aligned} |\varepsilon - \varepsilon'| &= \left| \sum_i a_i p_i - \sum_i a'_i p'_i \right| \\ &\leq \left| \sum_i p_i (a_i - a'_i) \right| + \left| \sum_i a'_i (p_i - p'_i) \right| \\ &\leq \sum_i p_i |a_i - a'_i| + \sum_i 4|p_i - p'_i| \\ &\leq \lambda \|p - p'\| + 4\|p - p'\| = (\lambda + 4)\|p - p'\|, \end{aligned}$$

because $0 \leq (f_{\mathscr{X}}(\boldsymbol{x}_i) - y_i)^2 \leq 4$ since $-1 \leq f_{\mathscr{X}}(\boldsymbol{x}) \leq 1$. □

Proof of Supplementary Lemma S3

Proof. Just plug in Lemma S1 into Theorem S5. □

Proof of Supplementary Theorem S6

Proof. Let ℓ be a B-Lipschitzian loss function with respect to its first variable and let $0 \leq \ell(\Phi_{\mathscr{X}}, z) \leq M$ for all $z \in \mathscr{Z}$. Therefore, if our subbagging algorithm has β_N pointwise hypothesis stability with respect to ℓ, then applying Theorem S3 yields the following upper bound for the generalization error of $\Phi_{\mathscr{X}}$:

$$R(\Phi_{\mathscr{X}}) \leq R_{emp}(\Phi_{\mathscr{X}}) + 2\beta_N + (4N\beta_N + M)\sqrt{\frac{\ln(1/\delta)}{2N}}.$$

From Proposition S1, the pointwise hypothesis stability β_N at ℓ is bounded above by

$$\beta_N \leq B\beta_p \frac{p}{N}.$$

The base machine used is β_p-stable Gentle Boost, where the value of β_p is obtained from Lemma S3. This completes the proof and the upper bound of $R(\Phi_{\mathscr{X}})$ follows immediately. □

Proof of Supplementary Theorem S7

Proof. Recall that Gentle Boost uses adaptive Newton steps to minimize $\mathbf{E}\left[e^{-y(F(\boldsymbol{x})+f(\boldsymbol{x}))}\right]$ exactly with respect to $f(\boldsymbol{x})$ [S2]. Since $f_t(\boldsymbol{x})$ is a weak learning algorithm, it follows that

$$\sum_{i=1}^{p} y_i f_t(\boldsymbol{x}_i) \geq 0. \tag{35}$$

Then,

$$\begin{aligned} e^{-\sum_{i=1}^{p} y_i f_t(\boldsymbol{x}_i)} &\leq 1 \\ e^{-\sum_{i=1}^{p} y_i F(\boldsymbol{x}_i)} e^{-\sum_{i=1}^{p} y_i f_t(\boldsymbol{x}_i)} &\leq e^{-\sum_{i=1}^{p} y_i F(\boldsymbol{x}_i)} \\ \prod_{i=1}^{p} e^{-y_i F(\boldsymbol{x}_i)} \prod_{i=1}^{p} e^{-y_i f_t(\boldsymbol{x}_i)} &\leq \prod_{i=1}^{p} e^{-y_i F(\boldsymbol{x}_i)} \\ \prod_{i=1}^{p} e^{-y_i (F(\boldsymbol{x}_i) + f_t(\boldsymbol{x}_i))} &\leq \prod_{i=1}^{p} e^{-y_i F(\boldsymbol{x}_i)}. \end{aligned}$$

It immediately follows that

$$\sum_{i=1}^{p} e^{-y_i (F(\boldsymbol{x}_i) + f_t(\boldsymbol{x}_i))} \leq \sum_{i=1}^{p} e^{-y_i F(\boldsymbol{x}_i)}, \tag{36}$$

since e^{-x} is monotonically decreasing and $e^{-x} > 0$ over the reals. □

Proof of Supplementary Theorem S8

Proof. First, up to round t inclusive,

$$\begin{aligned} R_{emp}^{W-CLB}(F^h_{t,\mathscr{X}'}, \mathscr{X}') &= \frac{1}{p}\left[e^{-y_i F_{t-1,\mathscr{X}}(\boldsymbol{x}_i)} e^{-y'_i f^h_{t,\mathscr{X}'}(\boldsymbol{x}'_i)} + \sum_{k \neq i} e^{-y_k (F_{t-1,\mathscr{X}}(\boldsymbol{x}_k) + f^h_{t,\mathscr{X}'}(\boldsymbol{x}_k))}\right] \\ &\leq R_{emp}(F_{t,\mathscr{X}}, \mathscr{X}), \end{aligned}$$

according to Equation (22), Algorithm 4, and Equation (24), i.e.,$e^{-y'_i f^h_{t,\mathscr{X}'}(\boldsymbol{x}'_i)} \leq e^{-y_i f_{t,\mathscr{X}}(\boldsymbol{x}_i)}$. Thus, we can apply Theorem S7, because the weights $w_{1,t+1}, \ldots, w_{p,t+1}$ form a probability distribution as a result of the modified normalization constant $Z'_t \leq Z_t$ (we still have boosting by definition). It follows that

$$R_{emp}^{W-CLB}(F^h_{t,\mathscr{X}'} + f_{t+1,\mathscr{X}'}, \mathscr{X}') \leq R_{emp}^{W-CLB}(F^h_{t,\mathscr{X}'}, \mathscr{X}') \leq R_{emp}(F_{t,\mathscr{X}}, \mathscr{X}), \tag{37}$$

and with probability of ω it follows that

$$R_{emp}^{W-CLB}(F^h_{t,\mathscr{X}'} + f_{t+1,\mathscr{X}'}, \mathscr{X}') \leq R_{emp}(F_{t,\mathscr{X}} + f_{t+1,\mathscr{X}}, \mathscr{X}). \tag{38}$$

□

Proof of Supplementary Proposition S3

Proof. We prove Equation (30) by analyzing the first-order partial derivative of the absolute loss difference by $yf(\boldsymbol{x})$.

Let ε denote the deviation of the margin $y_i f(\boldsymbol{x}_i)$ when the corresponding instance $z_i \in \mathscr{X}$ is replaced by $z \in \mathscr{X}$, or

$$y_i f_{\mathscr{X}^{\setminus i \cup z}}(\boldsymbol{x}_i) = y_i f_{\mathscr{X}}(\boldsymbol{x}_i) + \varepsilon, \quad -1 \leq \varepsilon \leq 1, \varepsilon \neq 0, \varepsilon \in \mathbb{R}.$$

Henceforth,

$$\begin{aligned}\frac{\partial}{\partial yf(\boldsymbol{x})}|e^{-yf(\boldsymbol{x})} - e^{-(yf(\boldsymbol{x})+\varepsilon)}| &= \frac{e^{-yf(\boldsymbol{x})} - e^{-(yf(\boldsymbol{x})+\varepsilon)}}{|e^{-yf(\boldsymbol{x})} - e^{-(yf(\boldsymbol{x})+\varepsilon)}|}\frac{\partial}{\partial yf(\boldsymbol{x})}(e^{-yf(\boldsymbol{x})} - e^{-(yf(\boldsymbol{x})+\varepsilon)}) \\ &= \frac{-e^{-2yf(\boldsymbol{x})}(1-e^{-\varepsilon})^2}{|e^{-yf(\boldsymbol{x})} - e^{-(yf(\boldsymbol{x})+\varepsilon)}|} < 0, \end{aligned} \tag{39}$$

since the exponential function is positive over $\mathbb{R}$. Equation (39) is true regardless of ε, or more precisely, regardless of whether ε reduces, increases or even flips the sign of the margin $yf(x)$. □

Proof of Supplementary Theorem S9

Proof. Let $\Phi_{\mathscr{X}}$ be the outcome of a real-valued Subbagged Gentle Boost model, trained on a training set $\mathscr{X}$ of size $N \geq 1$, that has a uniform (resp. hypothesis and pointwise hypothesis) stability β_N^u with respect to a loss function ℓ such that $0 \leq \ell(\Phi_{\mathscr{X}}, z) \leq M$, for all $z \in \mathscr{X}$. According to Theorem S3, for any $\delta \in (0,1)$, with probability at least $1-\delta$ over the random draw of $\mathscr{X}$, the generalization error of the overall subbagged model $R(\Phi_{\mathscr{X}}, \mathscr{X})$ is bounded from above by

$$R(\Phi_{\mathscr{X}}, \mathscr{X}) \leq R_{emp}(\Phi_{\mathscr{X}}, \mathscr{X}) + 2\beta_N^u + (4N\beta_N^u + M)\sqrt{\frac{ln(1/\delta)}{2N}}.$$

Now, if ℓ is a classification loss function, i.e.,$\ell(\Phi_{\mathscr{X}}, z) = \ell_\gamma(\Phi_{\mathscr{X}}, z), \forall z \in \mathscr{X}$, then by plugging Lemma S2 into the previous expression and considering the fact that ℓ_γ is bounded by $M = 1$, the corresponding upper bound based on the classification loss is

$$R^\gamma(\Phi_{\mathscr{X}}, \mathscr{X}) \leq R^\gamma_{emp}(\Phi_{\mathscr{X}}, \mathscr{X}) + 2\frac{\beta_N^c}{\gamma} + (4N\frac{\beta_N^c}{\gamma} + 1)\sqrt{\frac{ln(1/\delta)}{2N}},$$

where R^γ and R^γ_{emp} represent the adequate error estimates with respect to ℓ_γ, while β_N^c denotes the model's classification stability. In addition, from the proof of Theorem 17[S5], we know that regardless of the loss measure choice, the loss-independent generalization error $R(\Phi_{\mathscr{X}}, \mathscr{X}) \leq R^\gamma(\Phi_{\mathscr{X}}, \mathscr{X}) = \mathbf{E}_z\left[\ell_\gamma(\Phi_{\mathscr{X}}, z)\right], \forall z \in \mathscr{X}$.

In order to provide an upper bound on $R(\Phi_{\mathscr{X}}, \mathscr{X})$ that is going to hold regardless of the model's stability measure type, a simplified and more convenient upper bound expression is needed. The most straight-forward way to achieve this is to choose γ such that $\beta_N^u = \beta_N^c = \beta_N$. This can be done by simply choosing $\gamma = 1$. Consequently, it follows that

$$R(\Phi_{\mathscr{X}}, \mathscr{X}) \leq R^1_{emp}(\Phi_{\mathscr{X}}, \mathscr{X}) + 2\beta_N + (4N\beta_N + 1)\sqrt{\frac{ln(1/\delta)}{2N}},$$

where β_N is now the stability of $\Phi_{\mathscr{X}}$ with respect to ℓ_1.

The main limitation of the bound presented above is the fact that both $R^1_{emp}(\Phi_{\mathscr{X}}, \mathscr{X})$ and β_N are based on the ℓ_1 loss measure. Obviously, the ℓ_1 measure's output is a true representative of the loss of a real-valued algorithm A with respect to an instance-label pair $z = (\boldsymbol{x}, y)$ when its margin with respect to A falls between 0 and 1. But, since the output of a Gentle Boost ensemble of T weak learners ranges from $-T$ to T, the values of $yF_{\mathscr{X}^{(j)}}(\boldsymbol{x})$ will fall in the same range, for each $j = 1, \ldots, S$. Consequently, the corresponding margins $y\Phi_{\mathscr{X}}(\boldsymbol{x}) = \frac{1}{S}\sum_{j=1}^S yF_{\mathscr{X}^{(j)}}(\boldsymbol{x}) \in [-T, T], \forall z = (\boldsymbol{x}, y) \in \mathscr{X}$. Therefore, a more suitable way to measure the loss of the whole model and its constituent ensembles is to use the ℓ_T classification loss function. So, let β_p denote the stability of the model's base machine with respect to ℓ_T. We consider the fact that ℓ_1 is 1-Lipschitzian w.r.t. its first variable $\Phi_{\mathscr{X}}$, which was presented in the proof of Lemma S2. Thus, by applying Proposition S2, we obtain the following upper bound

$$R(\Phi_{\mathscr{X}}, \mathscr{X}) \leq R^1_{emp}(\Phi_{\mathscr{X}}, \mathscr{X}) + 4\beta_p\frac{p}{N} + (8N\beta_p\frac{p}{N} + 1)\sqrt{\frac{ln(1/\delta)}{2N}},$$

where p is the size of each data subset $\mathscr{X}^{(j)}$ used to train a single base machine, i.e.,$p = |\mathscr{X}^{(j)}|$, for each $j = 1, \ldots, S$.

At last, the difference between the values of both classification loss measures

$$\ell_1(\Phi_{\mathscr{X}},z)-\ell_T(\Phi_{\mathscr{X}},z)=\begin{cases}0, & \text{if } y\Phi_{\mathscr{X}}(\boldsymbol{x})\leq 0\\ (1-y\Phi_{\mathscr{X}}(\boldsymbol{x}))-\left(1-\frac{y\Phi_{\mathscr{X}}(\boldsymbol{x})}{T}\right), & otherwise\end{cases}$$

$$=\begin{cases}0, & \text{if } y\Phi_{\mathscr{X}}(\boldsymbol{x})\leq 0\\ y\Phi_{\mathscr{X}}(\boldsymbol{x})\frac{1-T}{T}, & otherwise\end{cases}, \quad \forall z\in\mathscr{Z}.$$

Due to the boosting nature of the underlying ensembles, they must be composed of at least two base learners, i.e.,$T>1$ must be satisfied. With this being taken into account,

$$\ell_1(\Phi_{\mathscr{X}},z)-\ell_T(\Phi_{\mathscr{X}},z)\leq 0$$

$$\ell_1(\Phi_{\mathscr{X}},z)\leq \ell_T(\Phi_{\mathscr{X}},z), \quad \forall z\in\mathscr{Z}.$$

Consequently,

$$\ell_1(\Phi_{\mathscr{X}},z_i)\leq \ell_T(\Phi_{\mathscr{X}},z_i), \quad \forall i\in\{1,\dots,N\}$$

$$\frac{1}{N}\sum_{i=1}^{N}\ell_1(\Phi_{\mathscr{X}},z_i)\leq \frac{1}{N}\sum_{i=1}^{N}\ell_T(\Phi_{\mathscr{X}},z_i)$$

$$R^1_{emp}(\Phi_{\mathscr{X}},\mathscr{X})\leq R^T_{emp}(\Phi_{\mathscr{X}},\mathscr{X})$$

$$\Longrightarrow R(\Phi_{\mathscr{X}},\mathscr{X})\leq R^T_{emp}(\Phi_{\mathscr{X}},\mathscr{X})+4\beta_p\frac{p}{N}+(8N\beta_p\frac{p}{N}+1)\sqrt{\frac{ln(1/\delta)}{2N}}.$$

Given the fraction $\eta=p/N$, after replacing it in the previous expression, we get the resulting upper bound

$$R(\Phi_{\mathscr{X}},\mathscr{X})\leq R^T_{emp}(\Phi_{\mathscr{X}},\mathscr{X})+4\eta\beta_p+(8\eta N\beta_p+1)\sqrt{\frac{ln(1/\delta)}{2N}}.$$

□

Proof of Supplementary Theorem S10

Proof. Let us assume that the j-th and the k-th Gentle Boost ensemble within the Subbagged Gentle Boost model are about to collaborate at the τ-th iteration of S-CLB, where $\tau\in[0,\mathscr{T}-1]$. By obtaining the optimization in the third step of the τ-iteration, the training subsets of both ensembles are updated as

$$\mathscr{X}^{(s,\tau+1)}=\begin{cases}\mathscr{X}^{(s,\tau)}, & \text{if } \mathscr{S}^{(j,k,\tau)}_{p^*}=\varnothing\\ \mathscr{X}^{(s,\tau)}_{p^*}, & otherwise\end{cases}.$$

Case 1 ($\mathscr{S}^{(j,k,\tau)}_{p^*}=\varnothing$):

In this case, the empirical error of $\Phi_{\mathscr{X}}$, simply remains the same, i.e.,$R^T_{emp}(\Phi^{(\tau)}_{\mathscr{X}},\mathscr{X})=R^T_{emp}(\Phi^{(\tau+1)}_{\mathscr{X}},\mathscr{X})$.

Case 2 ($\mathscr{S}^{(j,k,\tau)}_{p^*}\neq\varnothing$):

It is clear that if the optimal swapping order $\mathscr{S}^{(j,k,\tau)}_{p^*}\neq\varnothing$, the indicator variable $I^{(j,k,\tau)}_{p^*}=1$. This can only be the case when

$$R^{(j,\tau)}_{p^*,diff}\geq 0\land R^{(k,\tau)}_{p^*,diff}\geq 0. \quad (40)$$

Taking the former into consideration, we get

$$R^{(j,\tau)}_{p^*,diff}=R^T_{emp}(F^{(\tau)}_{\mathscr{X}^{(j,\tau)}},\mathscr{X}^{(j,\tau)})-R^T_{emp}(F^{(\tau)}_{\mathscr{X}^{(j,\tau)}_{p^*}},\mathscr{X}^{(j,\tau)}_{p^*})\geq 0$$

$$R^T_{emp}(F^{(\tau)}_{\mathscr{X}^{(j,\tau)}},\mathscr{X}^{(j,\tau)})\geq R^T_{emp}(F^{(\tau)}_{\mathscr{X}^{(j,\tau)}_{p^*}},\mathscr{X}^{(j,\tau)}_{p^*}) \quad (41)$$

$$R^T_{emp}(F^{(\tau)}_{\mathscr{X}^{(j,\tau)}},\mathscr{X}^{(j,\tau)})\geq R^T_{emp}(F^{(\tau+1)}_{\mathscr{X}^{(j,\tau+1)}},\mathscr{X}^{(j,\tau+1)}).$$

Analogously, for the latter in (40), we have

$$R^T_{emp}(F^{(\tau)}_{\mathscr{X}^{(k,\tau)}}, \mathscr{X}^{(k,\tau)}) \geq R^T_{emp}(F^{(\tau+1)}_{\mathscr{X}^{(k,\tau+1)}}, \mathscr{X}^{(k,\tau+1)}). \tag{42}$$

By combining (41) and (42),

$$R^T_{emp}(F^{(\tau)}_{\mathscr{X}^{(j,\tau)}}, \mathscr{X}^{(j,\tau)}) + R^T_{emp}(F^{(\tau)}_{\mathscr{X}^{(k,\tau)}}, \mathscr{X}^{(k,\tau)}) \geq R^T_{emp}(F^{(\tau+1)}_{\mathscr{X}^{(j,\tau+1)}}, \mathscr{X}^{(j,\tau+1)}) + R^T_{emp}(F^{(\tau+1)}_{\mathscr{X}^{(k,\tau+1)}}, \mathscr{X}^{(k,\tau+1)}).$$

The j-th and the k-th ensemble are the only ones collaborating in the τ-th iteration, while the empirical errors of the rest do not change in the next iteration, i.e.,$R^T_{emp}(F^{(\tau)}_{\mathscr{X}^{(s,\tau)}}, \mathscr{X}^{(s,\tau)}) = R^T_{emp}(F^{(\tau+1)}_{\mathscr{X}^{(s,\tau+1)}}, \mathscr{X}^{(s,\tau+1)})$ for each $s = 1, \ldots, S$, $s \neq j, k$, hence the following holds

$$\sum_{s=1}^{S} R^T_{emp}(F^{(\tau)}_{\mathscr{X}^{(s,\tau)}}, \mathscr{X}^{(s,\tau)}) \geq \sum_{s=1}^{S} R^T_{emp}(F^{(\tau+1)}_{\mathscr{X}^{(s,\tau+1)}}, \mathscr{X}^{(s,\tau+1)}).$$

Just by multiplying both sides with $1/S$ and taking (28) into consideration, it immediately follows that

$$R^T_{emp}(\Phi^{(\tau)}_{\mathscr{X}}, \mathscr{X}) \geq R^T_{emp}(\Phi^{(\tau+1)}_{\mathscr{X}}, \mathscr{X}).$$

$\Longrightarrow$ So, from **Case 1 & 2**, it is obvious that regardless of whether the optimal swapping order is determined at iteration τ, it holds that the empirical error of the whole model will decrease or at least remain constant at iteration $\tau + 1$, for every $\tau = 0, \ldots, \mathscr{T} - 1$. According to this,

$$R^T_{emp}(\Phi^{(0)}_{\mathscr{X}}, \mathscr{X}) \geq R^T_{emp}(\Phi^{(1)}_{\mathscr{X}}, \mathscr{X}) \geq \cdots \geq R^T_{emp}(\Phi^{(\mathscr{T}-1)}_{\mathscr{X}}, \mathscr{X}) \geq R^T_{emp}(\Phi^{(\mathscr{T})}_{\mathscr{X}}, \mathscr{X}),$$

and therefore

$$R^T_{emp}(\Phi^{(\tau')}_{\mathscr{X}}, \mathscr{X}) \geq R^T_{emp}(\Phi^{(\tau'')}_{\mathscr{X}}, \mathscr{X}), \quad \forall \tau', \tau'' \in [0, \mathscr{T}], \tau' < \tau''.$$

□

Proof of Supplementary Proposition S4

Proof. Let $z_i \in \mathscr{X}$ is correctly classified by $F_{T,\mathscr{X}}$. Then for its margin with respect to $F_{T,\mathscr{X}}$, it holds that $y_i F_{T,\mathscr{X}}(\boldsymbol{x}_i) > 0$. Consequently, the absolute classification loss difference,

$$|\ell_T(F_{T,\mathscr{X}}, z_i) - \ell_T(F_{T,\mathscr{X}^{\setminus i \cup z}}, z_i)| = \left| 1 - \frac{y_i F_{T,\mathscr{X}}(\boldsymbol{x}_i)}{T} - \ell_T(F_{T,\mathscr{X}^{\setminus i \cup z}}, z_i) \right|.$$

We are going to examine the value of the above expression as T increases, regardless of whether z_i is correctly classified by $F_{T,\mathscr{X}^{\setminus i \cup z}}$ or not.

Case 1 ($y_i F_{T,\mathscr{X}^{\setminus i \cup z}}(\boldsymbol{x}_i) \leq 0$)**:**

$$\frac{\partial}{\partial T} |\ell_T(F_{T,\mathscr{X}}, z_i) - \ell_T(F_{T,\mathscr{X}^{\setminus i \cup z}}, z_i)| = \frac{\partial}{\partial T} \frac{|-y_i F_{T,\mathscr{X}}(\boldsymbol{x}_i)|}{T} = -\frac{|y_i F_{T,\mathscr{X}}(\boldsymbol{x}_i)|}{T^2} < 0.$$

Case 2 ($y_i F_{T,\mathscr{X}^{\setminus i \cup z}}(\boldsymbol{x}_i) > 0$)**:**

$$\begin{aligned} \frac{\partial}{\partial T} |\ell_T(F_{T,\mathscr{X}}, z_i) - \ell_T(F_{T,\mathscr{X}^{\setminus i \cup z}}, z_i)| &= \frac{\partial}{\partial T} \frac{|y_i F_{T,\mathscr{X}^{\setminus i \cup z}}(\boldsymbol{x}_i) - y_i F_{T,\mathscr{X}}(\boldsymbol{x}_i)|}{T} \\ &= -\frac{|y_i F_{T,\mathscr{X}}(\boldsymbol{x}_i) - y_i F_{T,\mathscr{X}^{\setminus i \cup z}}(\boldsymbol{x}_i)|}{T^2} < 0. \end{aligned}$$

□

References

[S1] Kuncheva, L. I. *Combining pattern classifiers: methods and algorithms* (John Wiley & Sons, 2004).

[S2] Friedman, J., Hastie, T., Tibshirani, R. *et al.* Additive logistic regression: a statistical view of boosting (with discussion and a rejoinder by the authors). *The annals of statistics* **28**, 337–407 (2000).

[S3] Cortes, C. & Vapnik, V. Support-vector networks. *Machine learning* **20**, 273–297 (1995).

[S4] Breiman, L. Bagging predictors. *Machine learning* **24**, 123–140 (1996).

[S5] Bousquet, O. & Elisseeff, A. Stability and generalization. *Journal of Machine Learning Research* **2**, 499–526 (2002).

[S6] Kearns, M. & Ron, D. Algorithmic stability and sanity-check bounds for leave-one-out cross-validation. *Neural Computation* **11**, 1427–1453 (1999).

[S7] Elisseeff, A., Evgeniou, T. & Pontil, M. Stability of randomized learning algorithms. *Journal of Machine Learning Research* **6**, 55–79 (2005).

[S8] Kutin, S. & Niyogi, P. The interaction of stability and weakness in adaboost. technical report tr-2001-30. *Computer Science Department, University of Chicago* (2001).

[S9] Schapire, R. E., Freund, Y., Bartlett, P. & Lee, W. S. Boosting the margin: A new explanation for the effectiveness of voting methods. *Annals of statistics* 1651–1686 (1998).

[S10] Andonova, S., Elisseeff, A., Evgeniou, T. & Pontil, M. A simple algorithm for learning stable machines. In *ECAI*, 513–517 (2002).

[S11] Gao, W. & Zhou, Z.-H. Approximation stability and boosting. In *International Conference on Algorithmic Learning Theory*, 59–73 (Springer, 2010).

[S12] Grove, A. J. & Schuurmans, D. Boosting in the limit: Maximizing the margin of learned ensembles. In *AAAI/IAAI*, 692–699 (1998).
